# Small extracellular vesicles encapsulating CCL2 from activated astrocytes induce microglial activation and neuronal apoptosis after traumatic spinal cord injury

**DOI:** 10.1186/s12974-021-02268-y

**Published:** 2021-09-12

**Authors:** Yuluo Rong, Chengyue Ji, Zhuanghui Wang, Xuhui Ge, Jiaxing Wang, Wu Ye, Pengyu Tang, Dongdong Jiang, Jin Fan, Guoyong Yin, Wei Liu, Weihua Cai

**Affiliations:** Department of Orthopaedics, First Affiliated Hospital of Nanjing Medical University, Nanjing, 210029 Jiangsu China

**Keywords:** Spinal cord injury, Astrocyte, Small extracellular vesicles, CCL2, Microglia, Neuron

## Abstract

**Background:**

Spinal cord injury (SCI) is a severe traumatic disease which causes high disability and mortality rates. The molecular pathological features after spinal cord injury mainly involve the inflammatory response, microglial and neuronal apoptosis, abnormal proliferation of astrocytes, and the formation of glial scars. However, the microenvironmental changes after spinal cord injury are complex, and the interactions between glial cells and nerve cells remain unclear. Small extracellular vesicles (sEVs) may play a key role in cell communication by transporting RNA, proteins, and bioactive lipids between cells. Few studies have examined the intercellular communication of astrocytes through sEVs after SCI. The inflammatory signal released from astrocytes is known to initiate microglial activation, but its effects on neurons after SCI remain to be further clarified.

**Methods:**

Electron microscopy (TEM), nanoparticle tracking analysis (NTA), and western blotting were applied to characterize sEVs. We examined microglial activation and neuronal apoptosis mediated by astrocyte activation in an experimental model of acute spinal cord injury and in cell culture in vitro.

**Results:**

Our results indicated that astrocytes activated after spinal cord injury release CCL2, act on microglia and neuronal cells through the sEV pathway, and promote neuronal apoptosis and microglial activation after binding the CCR2. Subsequently, the activated microglia release IL-1β, which acts on neuronal cells, thereby further aggravating their apoptosis.

**Conclusion:**

This study elucidates that astrocytes interact with microglia and neurons through the sEV pathway after SCI, enriching the mechanism of CCL2 in neuroinflammation and spinal neurodegeneration, and providing a new theoretical basis of CCL2 as a therapeutic target for SCI.

**Supplementary Information:**

The online version contains supplementary material available at 10.1186/s12974-021-02268-y.

## Introduction

Spinal cord injury (SCI) is a severe traumatic disease which causes high disability and mortality rates. The stages of SCI have distinct molecular pathological characteristics, and some molecular mechanisms have been explored [1]. According to current research, reactive astrocytes play a key role in the neuroinflammatory response. Reactive astrocytes are now understood to release a variety of molecules, including inflammatory cytokines, chemokines, and neurotoxic substances, which aggravate inflammation and affect nerve function [2]. Once activated, these glial cells secrete many cytokines, chemokines, and pro-inflammatory mediators, which in turn affect the cellular states of peripheral nerve cells and other glial cells, thus triggering a vicious cycle and ultimately leading to exaggerated and uncontrolled inflammation [3]. Increasing evidence indicates that astrocytes play a neuro-inflammatory role in CNS diseases, such as Alzheimer’s disease, Parkinson’s disease, and SCI [1, 4, 5]. However, few studies have examined the intercellular communication of astrocytes through sEVs after SCI.

C-C Motif Chemokine Ligand 2 (CCL2), a member of the G protein-coupled receptor family, is also known as monocyte chemoattractant protein-1 (MCP-1), a member of the C-C subtype chemokine family [6, 7]. CCL2 attracts and acts through cells expressing the chemokine C-C motif receptor 2 (CCR2), thereby regulating the migration and penetration of monocytes, T lymphocytes, and natural killer cells to the inflammatory area [8]. Previous studies have shown that mice lacking CCL2 exhibit delayed cell death, decreased microglial activation, altered expression of cell death molecules, and improved neurological recovery after CNS injury [9, 10]. Recent studies have shown that the CCL2/CCR2 signaling pathway is involved in SCI and promotes inflammation and neurons damage [11]. However, after SCI, whether CCL2 exerts biological effects through sEV paracrine signaling remains unclear.

sEVs are the smallest endocytosis-derived membrane-bound nanovesicles involved in complex intercellular communication systems [12]. Several studies have shown that sEVs play a pathophysiological role in spinal cord tissue injury and repair by maintaining communication between cells. In this study, we found that astrocytes activated after SCI release CCL2, which acts on microglia and neurons through the sEV pathway. We additionally found that the binding of CCR2 promotes the apoptosis of neuron cells and the activation of microglia. Subsequently, the activated microglia release IL-1β, which acts on neuronal cells, thus further aggravating their apoptosis. In summary, the current study elucidates that astrocytes interact with microglia and neurons through the sEV pathway after SCI, enriching the mechanism of CCL2 in neuroinflammation and spinal neurodegeneration, and providing a new theoretical basis of CCL2 as a therapeutic target for SCI.

## Materials and methods

### Microarray data

The GSE42828 dataset was downloaded from the Gene Expression Omnibus (GEO) database. After normalization, log_2_ transformation, and probe annotation, genes with |log_2_ fold changes| > 1.5 and adjusted *P*-values < 0.05 were identified as differentially expressed genes (DEGs).

### Primary neuron, microglia, and astrocyte culture

Primary cortical neuronal cultures were obtained from embryonic (E16–E18) Sprague–Dawley (SD) rats as previously described [13]. Briefly, after dissection and cutting of brain cortices in Dulbecco’s modified Eagle’s medium/nutrient mixture F-12 (DMEM/F12; Thermo Fisher Scientific, USA), neurons were dissociated by digestion with 0.25% trypsin–EDTA solution (Thermo Fisher Scientific, MA, USA). After termination of the reaction, centrifugation was performed for 5 min (1000 rmp/min) at 4 °C to acquire cell suspensions. After being counted, cells were seeded on poly l-lysine-coated culture plates and replaced with Neurobasal medium (Thermo Fisher Scientific) supplemented with 2% B27 (Gibco Laboratory, Grand Island, NY), 2-mM glutamine (Gibco) and 1% penicillin–streptomycin. Half of the culture medium was exchanged every 2 days.

Primary microglia were obtained as previously reported [14]. Briefly, the cerebral hemispheres were carefully cut into small pieces of 0.5–1 mm^3^. Then, the tissue was digested as above, centrifuged, and resuspended in DMEM/F12 (Gibco Laboratory, Grand Island, NY). After filtering through a 100-μm nylon mesh, the final single cell suspension was cultured in T75 flasks pre-coated with poly-l-lysine (Sigma–Aldrich) to obtain a primary mixed glial cell culture. After 14 days of culture in vitro, the microglia reached maturity. The mature microglia were separated by shaking at 200 rpm for 2 h at room temperature. The microglial supernatant was collected and cultured in 6-well or 24-well culture plates pre-coated with poly-l-lysine at 37°C and 5% CO_2_ in an incubator. The medium was changed every 3 days.

Primary astrocytes were obtained from primary mixed glial cell cultures prepared as described above. After culturing in vitro for 3 days, the confluent culture was shaken on a shaker at 200 rpm for 2 h to remove microglia. The mixed glial cell culture was then treated with an astrocyte culture medium and shaken again at 240 rpm for 6 h to remove oligodendrocyte precursor cells (OPC). The remaining astrocytes were used for further experiments.

### Isolation and identification of sEVs

The extraction method for sEVs in the cell culture medium was performed as previously described [15, 16]. After co-culturing of astrocytes with or without TNF-α for 24 h, the culture medium was replaced with sEV-depleted FBS-containing medium for an additional 48 h. The culture medium was collected and centrifuged at 300 g for 10 min, and this was followed by centrifugation at 2000 × g for 10 min at 4 °C. After centrifugation, the cell supernatant was sterilized with a 0.22-μm filter to remove whole cells and cellular debris. Afterward, an Amicon Ultra-15 Centrifuge Filter Unit (Millipore) was used to concentrate the supernatant (4000 g, 40 min) until the volume of the upper chamber was decreased to nearly 200 μL. For purification, the samples were placed on top of a 30% sucrose/D_2_O cushion in a sterile Ultra-ClearTM tube (Beckman Coulter, Brea, CA, USA) and ultracentrifuged at 100,000 g for 2 h at 4°C. Partially purified sEVs were recovered with an 18-G needle, diluted in PBS and centrifuged at 4000×g at 4 °C until the final volume reached 200 μL. The solution was stored at − 80°C until further experiments.

The method for extraction of sEVs in spinal cord tissue is as follows. An appropriate amount of spinal cord tissue was chopped until homogenization, and the homogenate was placed in a test tube containing 75 U/mL collagenase and Hibernate-E (at a ratio of 800 μL per 100 mg of the spinal cord) for 20 min at 37 °C. The subsequent steps performed on the obtained liquefied homogenate were as described above.

To observe the morphology of the acquired sEVs derived from activated astrocytes (Acti-sEVs), transmission electron microscopy (TEM; Tecnai 12; Philips, Best, The Netherlands) was used. Nanosizer^TM^ technology (NTA, Malvern Instruments, Malvern, UK) was used to evaluate the diameter distribution. Specific surface markers, including CD9, CD63, CD81, and TSG101 were also detected by western blotting.

### sEV uptake

To investigate the uptake of intravenously injected Acti-sEVs, we fluorescently labeled sEVs with DiO (Molecular Probes, Eugene, OR, USA) according to the manufacturer’s instructions. Briefly, 4 mg/mL DiO was added to the sEV solution and incubated for 15 min at 4 °C. After incubation, PBS was added, and the mixture was then washed three times with ultracentrifugation at 100,000 g for 1 h to remove the excessive dye. The DiO-sEVs were injected intravenously into model rats through the tail vein. After 2 h, the rats were anesthetized, and frozen spinal cord sections were prepared. Slices were then stained with DAPI and imaged under a fluorescence microscope.

### Rat SCI model and experimental groups

Adult male Sprague–Dawley rats (180–220 g) were provided by the Animal Center of Nanjing Medical University (Nanjing, Jiangsu, China). The experimental protocol adhered to the National Institutes of Health Laboratory Animal Care and Use Guidelines and was approved by the Ethics Committee of Nanjing Medical University. The model of spinal cord contusion injury was established with the improved Allen method, as previously described [17]. Briefly, all animals were deeply anesthetized with pentobarbital (50 mg/kg) by intraperitoneal injection and underwent a laminectomy to expose the T10 spinal cord. A 10-g rod (2.5 mm in diameter; C4p01–001; RWD Life Science Corp, Shenzhen, China) was dropped from a height of 12.5 mm onto the spinal cord. Subsequently, the incisions were cleaned and sutured in layers. The bladders were manually voided twice daily until bladder function was restored.

The rats were randomly divided into three groups (*n* = 8 per group): an SCI group, Acti-sEV group, and INCB3344 group. The SCI, Acti-sEV group rats were administered tail vein injections of physiological saline (PBS, 200 μL) and Acti-sEVs (100 μg total protein in 200 μL of PBS), and INCB3344 (1 mM, 25 μL/rat) was injected in the vicinity of the spinal cord injury via the intrathecal route in the INCB3344 group immediately after SCI.

### Electrophysiology

For the assessment of motor-evoked potentials (MEPs), rats were anesthetized with 10% chloral hydrate solution by intraperitoneal injection. A single square wave stimulus with a duration of 0.5 ms, an intensity of 0.5 mA, and an interstimulus interval of 1 Hz was applied at the rostral ends of the exposed spinal cord, according to previous literature [14, 18–20]. The active electrode was inserted into the quadriceps muscle belly, the reference electrode was placed into the distal tendon of muscle, and the ground electrode was placed subcutaneously in the tail. The peak-to-peak amplitude was measured to evaluate nerve conduction function.

### Assessment of locomotor capacity

Neurological function was measured with the Basso–Beattie–Bresnahan (BBB) exercise scale and an inclined plane test 1, 3, 7, 14, 21, and 28 after SCI. The BBB test has a score ranging from 0 (complete paraplegia) to 21 (normal performance). The inclined plane test was performed on a testing apparatus, as described in previous reports [21, 22]. The maximum degree at which each rat held its position for at least 5 s was recorded. All assessments were performed by two examiners blinded to the treatments.

### Footprint analysis

Gait behavior and motor coordination were assessed 28 days post-surgery. The forelimbs (blue) and hind paws (red) were coated with dyes of different colors, and the rats were placed on absorbent paper surrounded by a cage. The animals were encouraged to walk in a straight line so that representative images of gaits could be assessed.

### Western blotting analysis

Proteins were extracted from cells and tissues, and protein concentrations were measured with BCA assays. Equal amounts of protein were separated by sodium dodecyl sulfate polyacrylamide gel electrophoresis and transferred onto polyvinylidene difluoride membranes. Membranes were blocked with 5% bovine serum albumin for 1 h at room temperature and then incubated overnight at 4 °C with antibodies against CCR2 (1:1000, Serotec), Cleaved caspase-3 (1:1000, Cell Signal Technology), Bcl-2 (1:1000, Abcam), Bax (1:1000, Abcam), TNF-α (1:1000, Abcam), IL-1β (1:1000, Abcam), IL-6 (1:1000, Abcam), IL-1Ra (1:1000, Abcam), OX42 (1:1000, Serotec), and GAPDH (1:1000, Abcam). After the membranes were washed with Tris-buffered saline with Tween, they were incubated for 2 h at room temperature with HRP-conjugated secondary antibodies (1:2000, Thermo Fisher Scientific, USA). The bands were visualized with enhanced chemiluminescence reagent (Thermo Fisher Scientific, USA), and the quantification of target proteins was performed in ImageJ (National Institutes of Health, Bethesda, MD, USA).

### Cell apoptosis measurements with TUNEL staining or flow cytometry in vitro

Our previous study demonstrated that exposure to glutamate (Glu; 100 μM) can be used as an in vitro SCI model [16, 23]. Primary spinal neurons pretreated with Acti-sEVs or PBS were subjected to Glu treatment and then reacted with terminal deoxynucleotidyl transferase dUTP nick end labeling (TUNEL) staining solution (Roche, Basel, Switzerland) for 30 min in the dark. After staining with DAPI (Beyotime Biotechnology, China) for 5 min, the cells were observed under a fluorescence microscope (AXIO Vert. A1 & Imager A2; Carl Zeiss Microscopy GmbH, Jena, Germany). TUNEL-positive (apoptotic) cells were counted in randomly selected fields of view in ImageJ (NIH, Bethesda, MD, USA).

The cell apoptotic rate was also examined with flow cytometry with an annexin V-FITC/PI apoptosis detection kit (BD Biosciences) according to the manufacturer’s instructions. After the indicated treatment, single-cell suspensions were harvested by centrifugation at 1500 rpm for 5 min and washed twice with cold PBS. Then, the cells were stained with 5 μl FITC-labeled annexin V and 5 μl PI in the dark for 5 min. The apoptosis rate was analyzed with flow cytometry (FACSVerse 8, BD Biosciences, Piscataway, NJ, USA).

### siRNA transfection

Small interfering RNAs targeting CCL2 and Rab27a (CCL2 siRNA and Rab27a siRNA), and their scrambled control siRNAs (NT siRNA), were purchased from GenePharma (Shanghai, China). A Lipofectamine 3000 reagent (Invitrogen) was used for transfection according to the manufacturer’s instructions.

### Quantitative real-time polymerase chain reaction

Total RNA was isolated with TRIzol reagent (Invitrogen) according to the manufacturer’s protocol. A PrimeScript RT reagent kit (TaKaRa, Japan) was used to synthesize cDNA from total RNA, and then, qRT-PCR was performed with a miScript SYBR Green qRT-PCR kit (QIAGEN, Germany) on an ABI 7900 fast real-time PCR system (Applied Biosystems, Carlsbad, USA). Expression levels were normalized to that of GAPDH, and the 2^−ΔΔCT^ method was used to analyze the relative gene expression. The specific primers were designed by RiboBio (Guangzhou, China) with the following sequences: TNF-α (forward: 5′-TCCCAGGTTCTCTTCAAGGGA-3′, reverse: 5′-GGTGAGGAGCACGTAGTCGG-3′), IL-1β (forward: 5′-GCCTCGTGCTGTCGGACCCATAT-3′, reverse: 5′-TCCTTTGAGGCCCAAGGCCACA-3′), IL-6 (forward: 5′-AAAGAGTTGTGCAATGGCAATTCT-3′, reverse: 5′-AAGTGCATCATCGTTGTTCATACA-3′), CCL2 (forward: 5′-CTTCTGGGCCTGCTGTTCA-3′, reverse: 5′-CCAGCCTACTCATTGGGATCA-3′) and CCR2 (forward: 5′-AGAGGCGAAGGCAACAGTCG-3′, reverse: 5′-GCAGGGCCAATGTCTAGTCC-3′).

### ELISA

To evaluate the expression levels of TNF-α, IL-1β, and IL-6 in the injured spinal cord, we processed tissues with a homogenizer and centrifuged the samples at 10 000 g for 10 min. The supernatants were collected, and the cytokine concentration was measured with ELISA kits (R&D Systems, Wiesbaden, Germany).

In the microglial culture medium, the cytokine levels (CCL2, TNF-α, IL-1β, and IL-6) were then determined with an ELISA kit (R&D Systems, Wiesbaden, Germany) according to the manufacturer’s instructions. Absorbance at 450 nm was measured with a plate reader.

### Nitric oxide determination

We spread microglia (2 × 10^5^ cells/mL, 200 μl/well) on a 96-well plate, collected the supernatant after treatment, and used Griess reagent (Promega, Madison, Wisconsin, USA). In brief, the supernatant was mixed with an equal amount of Griess reagent and incubated at room temperature for 10 min. The absorbance was measured by spectrophotometry at 540 nm, and the NO concentration was inferred from the standard curve of sodium nitrite (NaNO_2_).

### Transwell assays

In brief, a total of 2 × 10^4^ BV2 microglia were placed in the upper chamber, and the lower chamber was filled with 600 μl DMEM containing 15% FBS with or without 100 μg/mL Acti-sEVs. After incubation for 24 h, the cells on the upper surface of the membrane were cleaned with cotton swabs, whereas the cells attached to the bottom surface were fixed and stained with crystal violet for 10 min. The BV2 migrated cells were photographed under a fluorescence microscope and analyzed in ImageJ software.

### Preparation of spinal cord slices

Anesthetized rats were transcardially perfused with 0.9% saline solution followed by paraformaldehyde (PFA; 4% w/v). Lesion spinal segments were removed, fixed in 4% paraformaldehyde for 24 h at 4 °C, and dehydrated in 15% and 30% sucrose solutions. Spinal cord tissues were then embedded in optimal cutting temperature compound, cut into 18-μm longitudinal sections, and stored at − 80 °C until further use.

### Histological analysis

Hematoxylin and eosin staining was performed in accordance with the standard protocol to measure the cavity area of injury in each group [24]. Briefly, slices were stained with alum hematoxylin, differentiated with 0.3% acid ethanol, and then stained with eosin Y. Samples were then dehydrated, cleaned, and packed. Images were captured under a light microscope.

### TUNEL staining of spinal cord sections

Apoptotic cells were identified and quantified with the TUNEL method according to the manufacturer’s protocol. Spinal cord sections were fixed, blocked, and costained with TUNEL and DAPI. The percentage of TUNEL-positive cells was counted under a fluorescence microscope.

### Immunofluorescence staining

Cells or spinal cord sections were fixed with 4% paraformaldehyde for 15 min, permeabilized in 0.2% Triton X-100 for 30 min, then blocked in 10% BSA for 1 h and finally incubated overnight at 4°C with primary antibodies against the following proteins: GFAP (1:1000, Abcam), NeuN (1:500, Abcam), OX42 (1:200, Serotec), MAP2 (1:500, Abcam), CD11b (1:200, Abcam), CCL2 (1:250, Serotec), CCR2 (1:50, Santa Cruz), and IL-1Ra (1:100, Abcam). After incubation, cells or slides were rinsed gently in PBS three times and then incubated with Alexa Fluor 488- and Alexa Fluor 594-conjugated secondary antibodies (1:200, Jackson ImmunoResearch, USA) for 2 h at room temperature. Finally, nuclei were counterstained with DAPI. Images were captured under a fluorescence microscope with equal exposure times.

### Fluorescence in situ hybridization

Fluorescence in situ hybridization was performed according to a previously described protocol [25]. Briefly, after the sections dried for 30 min at room temperature, they were rinsed three times with 1× sodium citrate (SSC) for 5 min each. Pre-hybridization buffer heated to 47 °C was pre-hybridized with the tissue for 20 min. At the same time, the probe was diluted in hybridization buffer and denatured at 60 °C for 10 min. The pre-hybridization buffer was removed, and the probe was hybridized to the tissue overnight (12–16 h) at the same temperature as the pre-hybridization step. After hybridization, the sections were washed with 4× SSC, 2× SSC, and 1× SSC solutions at the same temperature as the pre-hybridization step. Finally, the sections were rinsed three times with 1× PBS. After in situ hybridization, sections were incubated with the corresponding primary and secondary antibodies as described before. The sections were sealed and observed with laser confocal microscopy (Zeiss, Oberkochen, Germany, LSM 510).

### Statistical analyses

Statistical analysis was performed in IBM SPSS Statistics v17.0 and GraphPad 8.0.2. The experiments were repeated independently at least three times, and the results are expressed as mean ± standard deviation. *P*-values were calculated with the Student’s t-test and one-way ANOVA. A *P*-value less than 0.05 was considered statistically significant.

## Results

### Astrocytes release more sEVs containing CCL2 after traumatic SCI

**Fig. 1:**
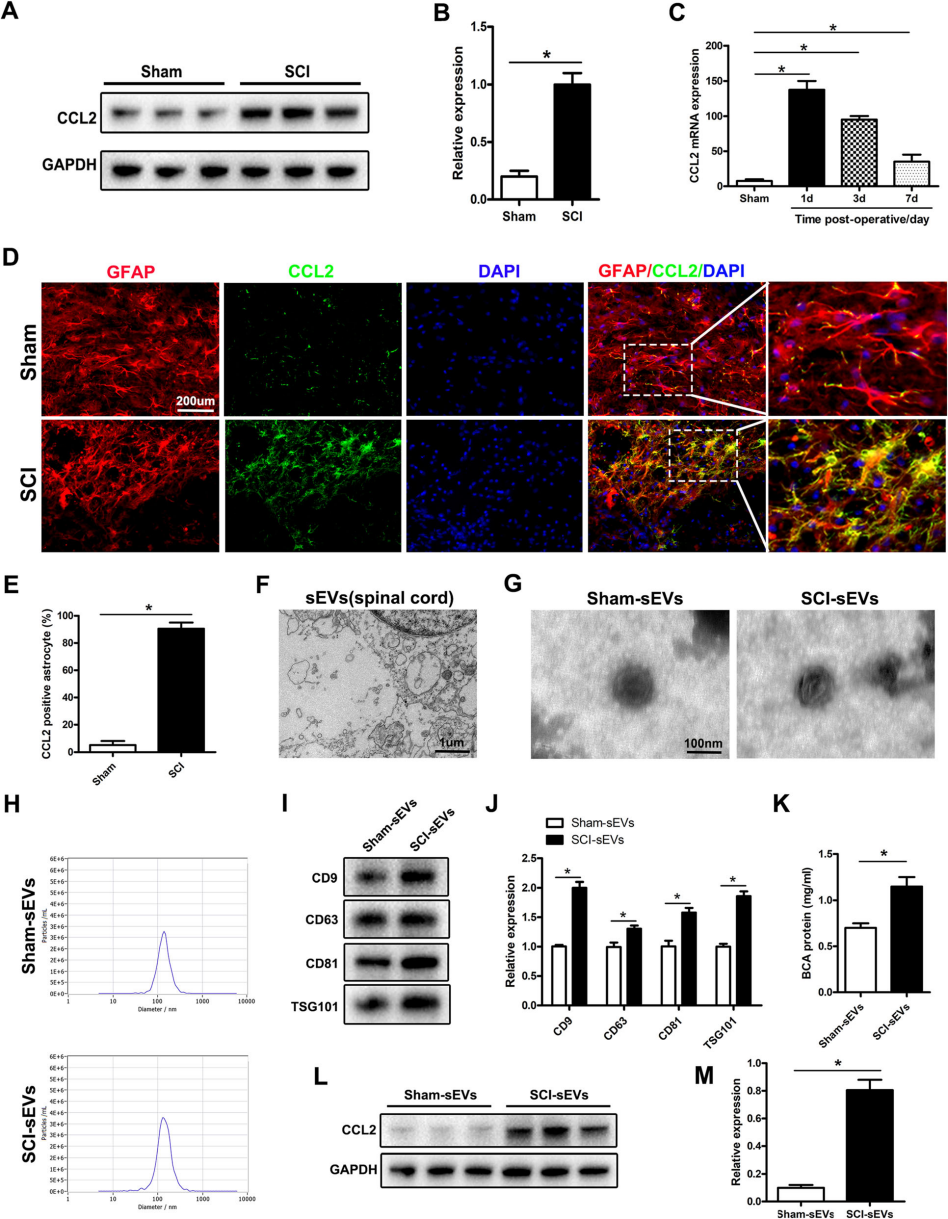
After traumatic SCI, astrocytes release more sEVs that encapsulate CCL2. **a**, **b** Western blotting to detect the expression of CCL2 in spinal cord tissue. **c** qRT-PCR to detect the expression of CCL2 in spinal cord tissue. **d**, **e** Immunofluorescence co-localization of astrocyte markers GFAP and CCL2. **f** TEM observation of sEVs released by cells in spinal cord tissue. **g** TEM observation of the typical structure of sEVs purified from spinal cord tissue. **h** NTA analysis of sham-sEVs and SCI-sEVs, showing that the small extracellular vesicles in the two groups had similar size ranges. **i**, **j** Western blotting analysis of sEV marker proteins TSG101, CD63, CD9 and CD81. **k** BCA method to determine the protein concentrations of the two groups of sEVs. **l**, **m** Western blotting to detect the expression of CCL2 in sEVs

After traumatic SCI, many released inflammatory cytokines are known to cause neuronal damage and loss of motor function. Through bioinformatics analysis, we found that the inflammatory factor CCL2 was released in large quantities after SCI (Supplementary Fig. 1). Therefore, we hypothesized that CCL2 might play an important role in SCI. Next, we used western blotting and qRT-PCR to detect the expression of CCL2 protein after SCI in rats. The results confirmed that CCL2 protein expression increased significantly at 1 day after SCI (Fig. 1a–c). Because previous studies have shown that CCL2 is expressed mainly in astrocytes, we tested the co-localization of the astrocyte marker GFAP and CCL2 protein. In the sham group, the expression of CCL2 in spinal cord tissue was very weak. However, 1 day after SCI, CCL2 was significantly upregulated and was mainly expressed in astrocytes (Fig. 1d, e). To understand the changes in the release of sEVs after SCI, we separated the spinal cord tissue and used TEM to reveal the sEV release from spinal cord tissue cells (Fig. 1f). We hypothesized that CCL2 released by astrocytes after SCI might affect other cells through paracrine signaling. To verify this hypothesis, we isolated and purified sEVs from spinal cord tissue and analyzed them with TEM, nanoparticle tracking analysis (NTA), and western blotting (using CD81, TSG101, CD9, and CD63 as sEV markers). The sEVs purified from spinal cord tissue showed typical sizes and shapes (Fig. 1g, h). SCI rats produced more sEVs in the spinal cord than rats in the sham group, on the basis of sEV-labeled protein quantification (Fig. 1i, j). In addition, compared with that in the normal spinal cord group, the sEV protein content in the injured spinal cord group was significantly higher (Fig. 1k). Moreover, compared with normal spinal cords, injured spinal cords secreted highly sEV-enriched CCL2 (Fig. 1l, m). In summary, these results indicated that astrocytes release more sEVs that contain CCL2 after SCI.

Given that CCL2 functions by binding its receptor, CCR2, we tested the expression of CCR2 protein after SCI. CCR2 protein expression was significantly higher in injured spinal cords than normal spinal cords (Supplementary Fig. 2A-C). Next, to define the cell distribution of CCR2, we performed fluorescence in situ hybridization (FISH) on spinal cord sections. The microglia marker CD11b and the neuron marker NeuN were used. We found that after spinal cord injury, CCR2 was expressed in microglia and neuronal cells (Supplementary Fig. 2D). Therefore, we speculated that after SCI, astrocytes released CCL2-encapsulating sEVs, which may be involved in microglial activation and neuronal apoptosis, although the specific mechanism of action requires further study.

### sEVs released by activated astrocytes induce microglial activation and neuronal apoptosis

**Fig. 2:**
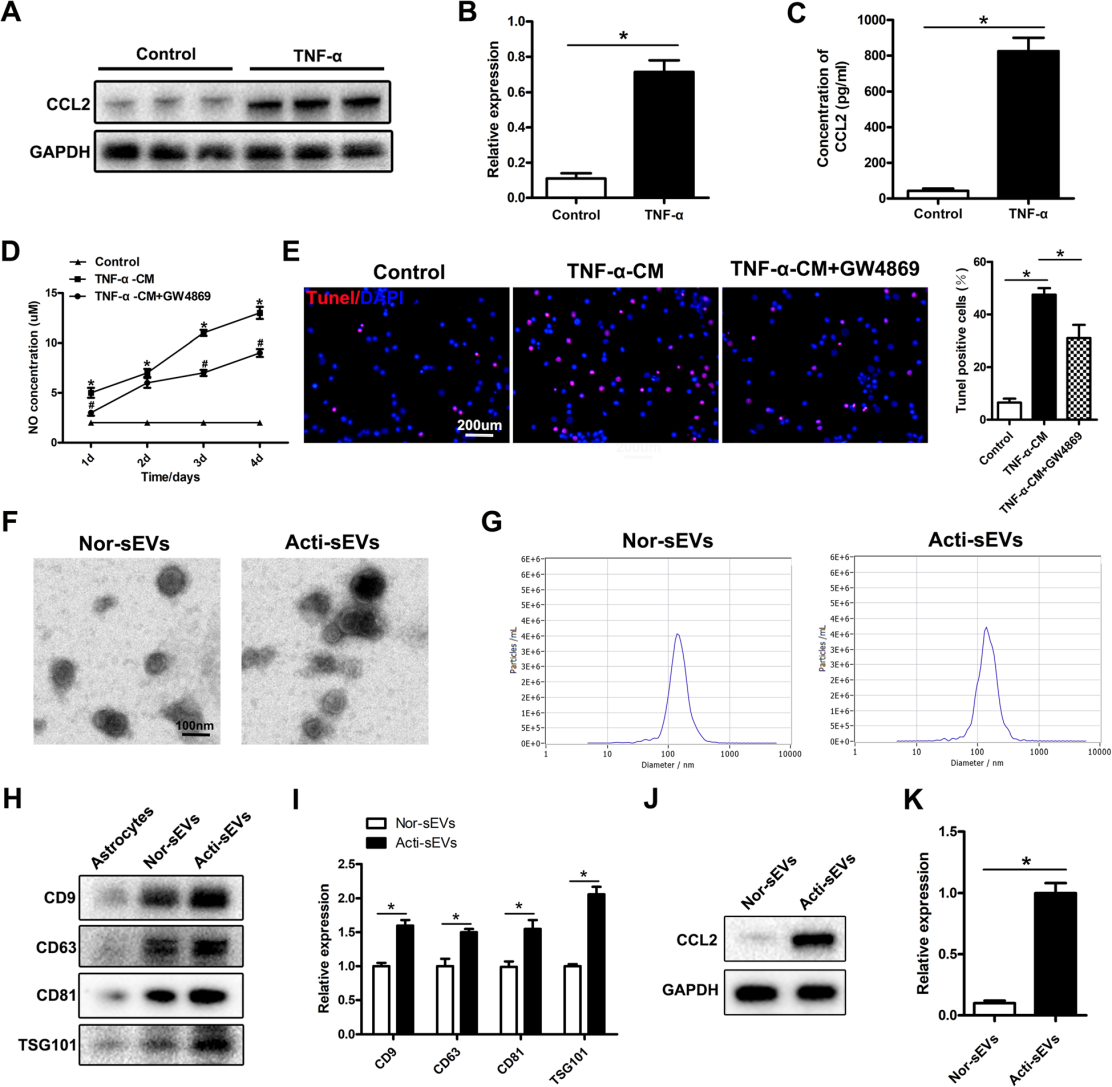
The effects of TNF-α-CM on microglial activation and neuronal apoptosis and the identification of sEVs. **a**, **b** Western blotting detection of CCL2 expression in astrocytes. **c** qRT-PCR to detect the expression of CCL2 in astrocytes. **d** Astrocyte culture medium stimulated by TNF-α (TNF-α-CM) promotes the release of NO from microglia, and the amount of NO released by microglia decreases after addition of an sEV inhibitor GW4869. **e** TUNEL staining to detect neuronal apoptosis. **f–i** TEM, NTA and western blotting to identify sEVs. **j**, **k** Western blotting to detect CCL2 expression in sEVs

To verify the effects of astrocyte CCL2 on microglial activation and neuronal apoptosis, we extracted primary astrocytes, microglia, and neuronal cells in vitro and identified them (Supplementary Fig. 3). As shown in Fig. 2a and b, astrocytes were incubated with TNF-α (10 ng/ml) for 15 min, and then the medium was replaced with a normal culture medium. Western blotting showed that the expression of CCL2 increased. The ELISA results further confirmed that activated astrocytes released large amounts of CCL2 (Fig. 2c). Next, to study the potential mechanisms of activated astrocytes in microglial activation and neuronal apoptosis, we co-cultured TNF-α-stimulated astrocyte culture medium (TNF-α-CM) with microglia. The TNF-α-CM promoted the release of NO from microglia, but interestingly, the amount of NO released by activation of microglia decreased after the addition of the sEV inhibitor GW4869 (St. Louis, MO, USA) (Fig. 2d). Therefore, we hypothesize that CCL2 is released through the sEV pathway after astrocyte activation, thus further verifying the results in vivo. Next, we investigated whether CCL2 might also affect neuronal apoptosis through the sEV pathway after astrocyte activation. TUNEL staining indicated that the addition of TNF-α-CM led to an increase in neuronal apoptosis, whereas neuronal apoptosis decreased after the addition of the sEV inhibitor GW4869, in agreement with our hypothesis (Fig. 2e). Rab27a is a key gene involved in regulating the release of sEVs [26]. Many studies have shown that knocking down Rab27a expression decreases sEV secretion [27, 28]. We further demonstrated, by knocking down the expression of astrocyte Rab27a, that inhibiting the release of activated astrocyte sEVs decreased the NO content released by microglial activation and neuronal cell apoptosis (Supplementary Fig. S4).

Next, to further clarify the involvement of sEVs containing CCL2 in microglial activation and neuronal apoptosis, we inoculated astrocytes with a normal culture medium and a TNF-α-stimulated astrocyte culture medium. After 48 h of culture, the sEVs were separated from the sEV-free serum medium through a combination of ultrafiltration and ultracentrifugation. The samples were then analyzed with TEM, NTA, and western blotting. TEM showed that in the normal medium and in the TNF-α-treated medium, the diameters of round nanoparticles ranged from 50 to 150 nm, and the size distribution in NTA was similar (Fig. 2f, g). No morphological differences were observed between groups in terms of size, shape, and electron density. Western blotting indicated the presence of sEV surface markers, including TSG101, CD9, CD63, and CD81 (Fig. 2h). After TNF-α stimulation of astrocyte activation, the protein levels of the sEV surface markers TSG101, CD9, CD63, and CD81 increased (Fig. 2h, i). The above results indicated that the release of sEVs after the activation of astrocytes increased. In addition, compared with the sEVs released by unactivated astrocytes (Nor-sEVs), those released after astrocyte activation were highly enriched in CCL2 protein (Fig. 2j, k), thus further indicating that the CCL2 released by activated astrocytes is encapsulated in sEVs.

**Fig. 3:**
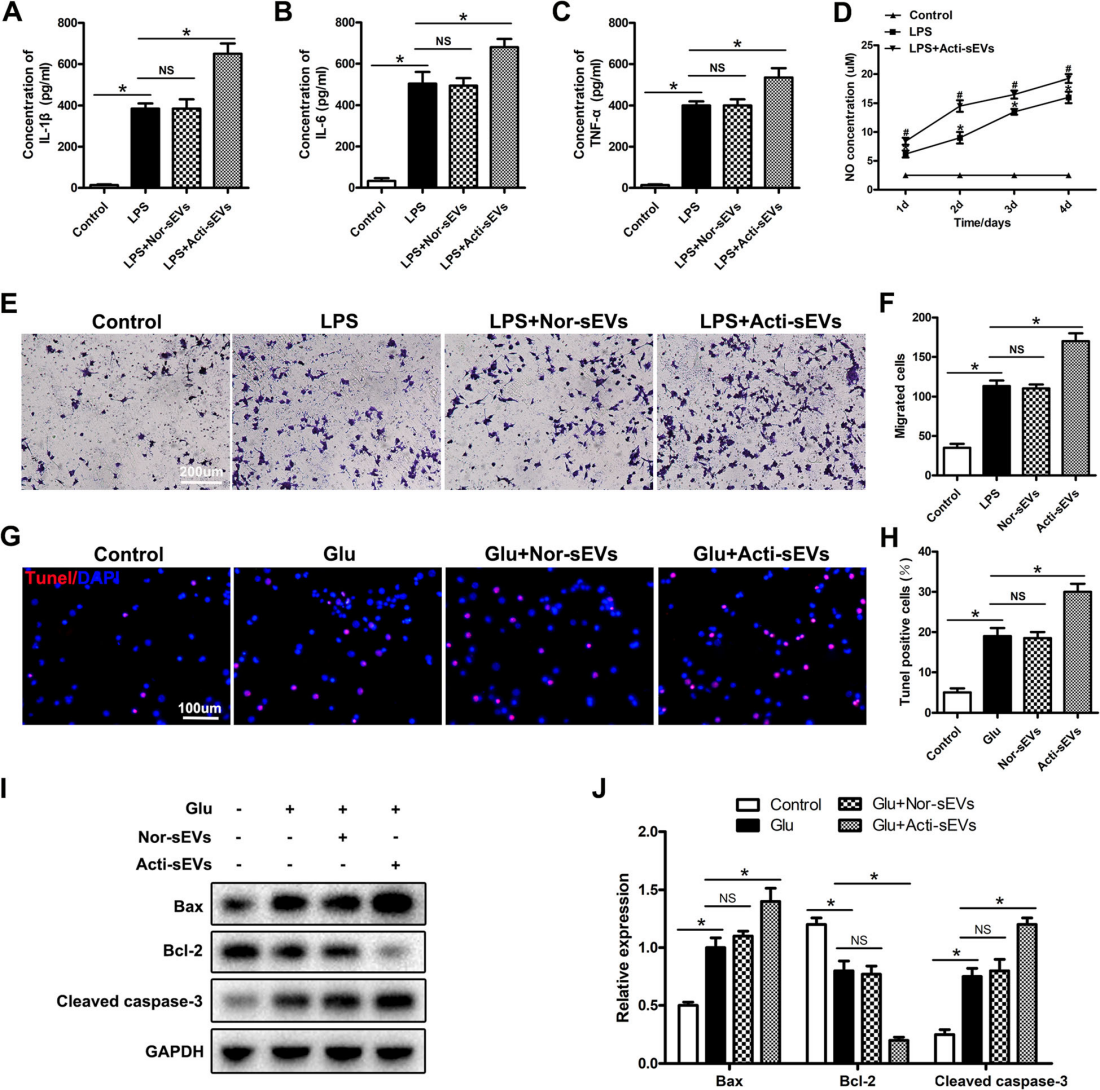
sEVs released by activated astrocytes induce microglial activation and neuronal apoptosis. **a**–**c** ELISA method to detect the expression of TNF-α, IL-1β, and IL-6. **d** Detection of changes in NO content. **e**, **f** Transwell assessment of the migration ability of BV2 microglia. **g**, **h** TUNEL staining detecting neuronal apoptosis. **i**, **j** Western blotting detecting the expression of apoptosis-related proteins in neurons

Next, we studied the effect of CCL2 encapsulated in sEVs on microglial activation and neuronal apoptosis. LPS is a potent inflammatory response inducer and a typical microglial activator. LPS binds the LPS receptor CD14 on the microglia membrane and subsequently induces microglial activation, thereby producing inflammatory mediators. Therefore, we used an LPS (1 μg/mL)-induced microglial activation model to simulate the activation state of microglia after SCI in vivo. Then we used Nor-sEVs or Acti-sEVs to culture LPS-treated microglia at a concentration of 100 μg/mL. The content of inflammatory factors released by activated microglia was examined with an ELISA kit. The ELISA results showed that the content of inflammatory factors released after LPS treatment increased. Compared with the results for the LPS group, Nor-sEV treatment did not increase the release of inflammatory factors; however, treatment with Acti-sEVs increased the release of inflammatory factors (Fig. 3a–c). Similar results were found by detecting changes in NO content (Fig. 3d). We then used Transwell assays to evaluate the effects of CCL2-encapsulating sEVs on the migration of BV2 microglia. Compared with the LPS group, the Acti-sEV group showed greater migration of microglia, whereas the Nor-sEV group showed no effect on the migration of microglia (Fig. 3e, f). Collectively, these results indicated that sEVs released by astrocytes induced microglial activation and migration.

High levels of extracellular glutamate (Glu) can cause neuronal cell death (excitatory toxicity), and Glu-induced toxicity is one of the most important pathogenic mechanisms of neuronal apoptosis and neurological dysfunction in spinal cord injury [29]. Therefore, we established a Glu-induced neuronal damage model to simulate neuronal cell damage in vivo. We cultured Glu-treated neuronal cells with Nor-sEVs or Acti-sEVs at a concentration of 100 μg/mL. TUNEL staining was used to evaluate the apoptosis of neuronal cells. Compared with the Glu group, the Acti-sEV treatment group showed greater neuronal apoptosis, whereas the neuronal apoptosis in the Nor-sEV group did not change (Fig. 3g, h). Western blotting further confirmed the results of TUNEL staining (Fig. 3i, j).

In summary, the above results indicated that sEVs containing CCL2 released by activated astrocytes induce microglial activation, migration and neuronal apoptosis.

### The effects of sEVs from astrocytes treated with CCL2 siRNA on microglial activation, migration and neuronal apoptosis

**Fig. 4:**
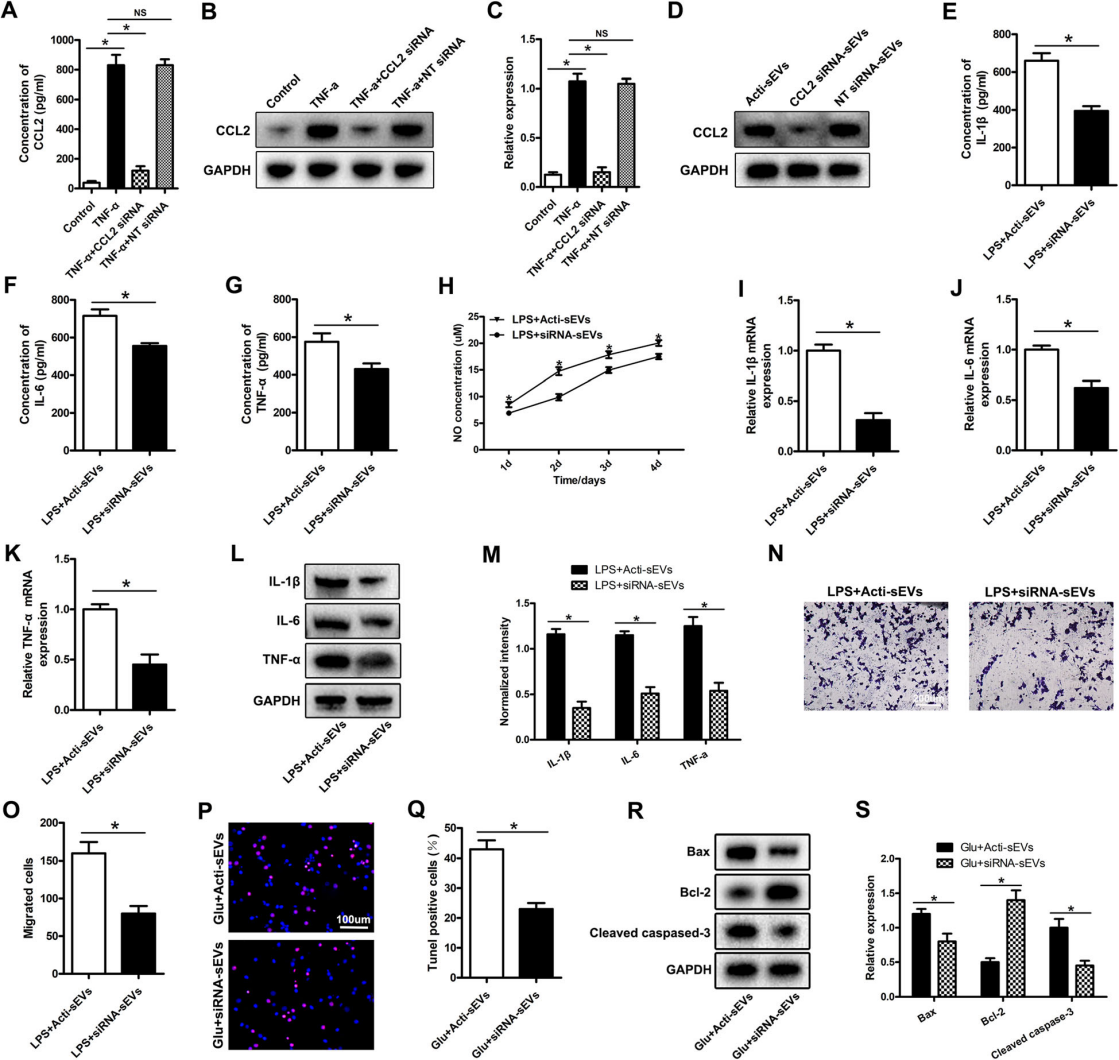
The effects of sEVs after CCL2 siRNA treatment of astrocytes on microglial activation, migration and neuronal apoptosis. **a–c** CCL2 expression and release detected by ELISA and western blotting. **d** Western blotting detection of CCL2 expression in sEVs. **e–g** ELISA method to evaluate the expression of TNF-α, IL-1β, and IL-6. **h** Detection of changes in NO content. **i–k** qRT-PCR to detect the expression of TNF-α, IL-1β, and IL-6. **l**, **m** Western blotting to detect the expression of pro-inflammatory related proteins TNF-α, IL-1β, and IL-6. **n**, **o** Transwell assessment of the migration ability of BV2 microglia. **p**, **q** TUNEL staining to detect neuronal apoptosis. **r**, **s** Western blotting to detect the expression of neuron apoptosis-related proteins

To further confirm the roles of CCL2 encapsulated in sEVs released by astrocytes in inducing microglial activation and neuronal apoptosis, we treated cultured astrocytes with short interfering RNAs targeting CCL2 (siRNA). The astrocytes were incubated with CCL2 siRNA for 24 h and then stimulated with TNF-α for 15 min. ELISA and western blotting results showed that CCL2 siRNA treatment inhibited the expression and release of CCL2 induced by TNF-α. Compared with untreated astrocytes, astrocytes treated with non-targeting siRNA showed no effect on CCL2 expression and release (Fig. 4a–c). Then we collected the medium supernatant, extracted sEVs and detected the expression of CCL2 in sEVs by western blotting. The expression of CCL2 in sEVs treated with CCL2 siRNA was lower than that in activated astrocyte sEVs (Fig. 4d). After successfully confirming that CCL2 expression and release were silenced in activated astrocyte sEVs, we evaluated the effect of this knockdown on microglial activation and migration. After being incubated with CCL2 siRNA (2 μg/ml, 24 h), astrocytes were stimulated with TNF-α for 15 min and washed with PBS. After 3 h, the culture medium was collected to extract sEVs and then added to the LPS-treated microglia culture. CCL2 siRNA-sEV treatment decreased the release of TNF-α, IL-1β, and IL-6 inflammatory factors and the NO content caused by Acti-sEVs (Fig. 4e–h). qRT-PCR and western blotting further confirmed the above ELISA results. CCL2 siRNA-sEV treatment decreased the expression of TNF-α, IL-1β, and IL-6 caused by Acti-sEVs (Fig. 4i–m). Transwell assays were used to evaluate the effects of CCL2 siRNA-sEVs on the migration of BV2 microglia. Compared with the Acti-sEV group, the CCL2 siRNA-sEV group showed a decrease in the migration of microglia (Fig. 4n, o). These results indicated that sEVs from CCL2 siRNA-treated astrocytes inhibited the activation and migration of microglia. Next, we evaluated the effect of CCL2 siRNA-sEVs on neuronal apoptosis. TUNEL staining results indicated that, compared with the Acti-sEV group, the CCL2 siRNA-sEV group showed less neuronal apoptosis (Fig. 4p, q), and western blotting further confirmed the TUNEL staining results (Fig. 4r, s).

In summary, the above results indicated that CCL2 siRNA treatment inhibits the effects of activated astrocytes on microglial activation, migration, and neuronal apoptosis.

### The effects of sEVs containing CCL2 on microglial activation and neuronal apoptosis after inhibition of CCR2

To confirm the role of the CCL2 receptor CCR2 in astrocyte-induced microglial activation, migration, and neuronal apoptosis, we added the specific CCR2 inhibitor INCB3344 (MCE, 10 nM) before LPS and Acti-sEVs were used to treat microglia. INCB3344 treatment decreased the release of TNF-α, IL-1β, and IL-6 and the increase in NO content caused by Acti-sEV treatment (Supplementary Fig. 5A–D). qRT-PCR and western blotting further confirmed the above ELISA results. INCB3344 treatment also decreased the expression of TNF-α, IL-1β, and IL-6 caused by Acti-sEV treatment (Supplementary Fig. 5E–H). Transwell assays were used to evaluate the effect of INCB3344 on the migration of BV2 microglia. Compared with the Acti-sEV group, the INCB3344 group showed less migration of microglia (Supplementary Fig. 5I, J). These results indicated that treatment with INCB3344 inhibited the activation and migration of microglia caused by activated astrocyte sEVs. Next, we evaluated the effect of Acti-sEVs on neuronal apoptosis after adding INCB3344. TUNEL staining showed that after the addition of INCB3344, Acti-sEVs decreased neuronal apoptosis (Supplementary Fig. 5K, L), and western blotting further confirmed the results of TUNEL staining (Supplementary Fig. 5M, N).

In summary, the above results indicated that INCB3344 weakens the effect of Acti-sEVs on microglial activation, migration and neuronal apoptosis.

### sEVs containing CCL2 aggravate microglial activation and neuronal apoptosis in vivo

**Fig. 5:**
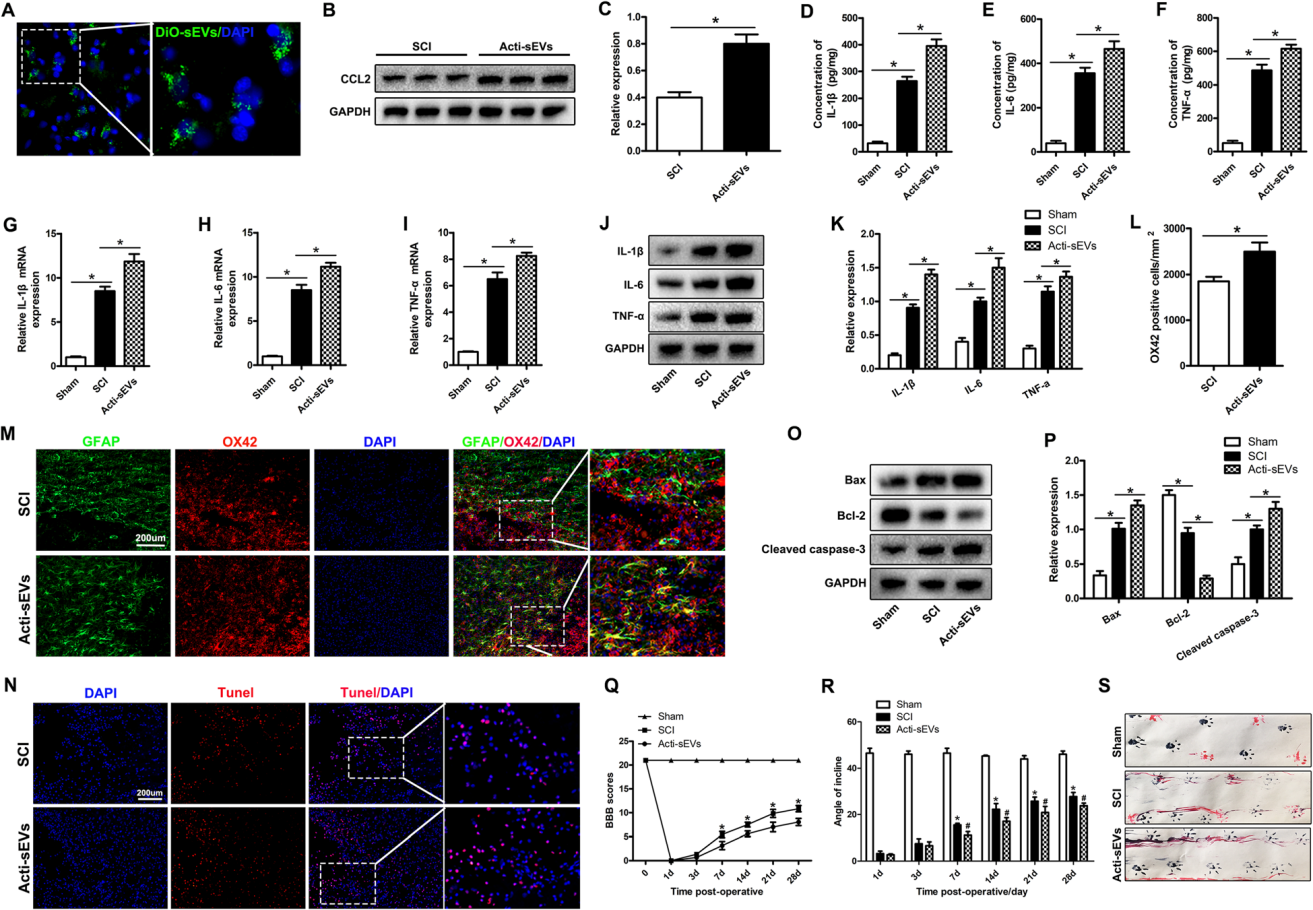
sEVs containing CCL2 from astrocytes aggravate microglial activation and neuronal apoptosis in vivo. **a** DiO-sEVs can enter the spinal cord tissue and be absorbed by cells. **b**, **c** Western blotting detection of spinal cord tissue CCL2 expression. **d–i** ELISA and qRT-PCR to evaluate the expression of TNF-α, IL-1β, and IL-6. **j**, **k** Western blotting detection of TNF-α, IL-1β, and IL-6 protein expression. **l**, **m** Immunofluorescence staining to detect the expression of the microglial activation marker OX42. **n** TUNEL staining to detect apoptosis of spinal cord tissue. **o**, **p** Western blotting to detect the expression of apoptosis-related proteins in spinal cord tissue. **q**, **r** BBB scores and inclined plane test at different times after SCI. **s** Representative footprints of rats walking 28 days after SCI

To confirm in vivo that the CCL2-encapsulating sEVs released by activated astrocytes aggravated the activation of microglia and neuronal apoptosis, we injected the encapsulated CCL2 sEVs released by activated astrocytes extracted in vitro into SCI rats. First, DiO-labeled Acti-sEVs were injected into rats through the tail vein to detect whether Acti-sEVs could enter the SCI site. As shown in Fig. 5a, under a fluorescence microscope, DiO-sEVs was found to accumulate at the SCI site and to be absorbed by cells after injection. Western blotting showed that after injection of Acti-sEVs, the expression of CCL2 protein in spinal cord tissue increased (Fig. 5b, c). Next, we used ELISA, qRT-PCR and western blotting to detect the changes in TNF-α, IL-1β, and IL-6 levels after activation of microglia. Compared with the SCI group, the Acti-sEV group showed higher expression of TNF-α, IL-1β, and IL-6 after SCI (Fig. 5d–f), and qRT-PCR and western blotting further confirmed the above results (Fig. 5g–k). The immunofluorescence results for the microglial activation marker OX42 showed that, compared with the SCI group, the Acti-sEV group showed greater activation of microglia after SCI (Fig. 5l, m). Then, the TUNEL method was used to evaluate neuronal cell apoptosis in the SCI area. Compared with the SCI group, the Acti-sEV group showed greater neuronal apoptosis (Fig. 5n). Western blotting results showed that the expression levels of pro-apoptotic markers Bax and Cleaved caspase-3 were higher, whereas that of the anti-apoptotic protein Bcl-2 was lower (Fig. 5o, p), in the Acti-sEV group than the SCI group, thus further confirming the results of TUNEL staining.

Next, we evaluated the effect of Acti-sEVs on motor function in SCI rats. The BBB score results showed that the recovery of the rats’ motor function gradually improved 1 week after SCI injury. At 2–4 weeks after SCI, the BBB score of the Acti-sEV group had recovered more slowly than that of the SCI group (Fig. 5q). The inclined plane test results also showed that the maximum inclination angle of the Acti-sEV group was lower than that of the SCI group (Fig. 5r). In addition, we performed behavioral tests on animals 28 days after SCI. Specifically, the walking pattern (gait) was evaluated through manual analysis of the footprints. After SCI, the coordination of fore-paw step movement in all animals decreased significantly. Compared with the animals in the SCI group, the animals in the Acti-sEV group showed slower gait recovery and improved motor coordination. The performance of the rats in the sham group remained unchanged throughout the test period (Fig. 5s).

In summary, the above results indicated that the CCL2-encapsulating sEVs released by activated astrocytes aggravate the activation of microglia and neuronal apoptosis in vivo, and weaken the recovery of motor function in SCI rats.

### CCL2-induced microglial activation may aggravate neuronal apoptosis

**Fig. 6:**
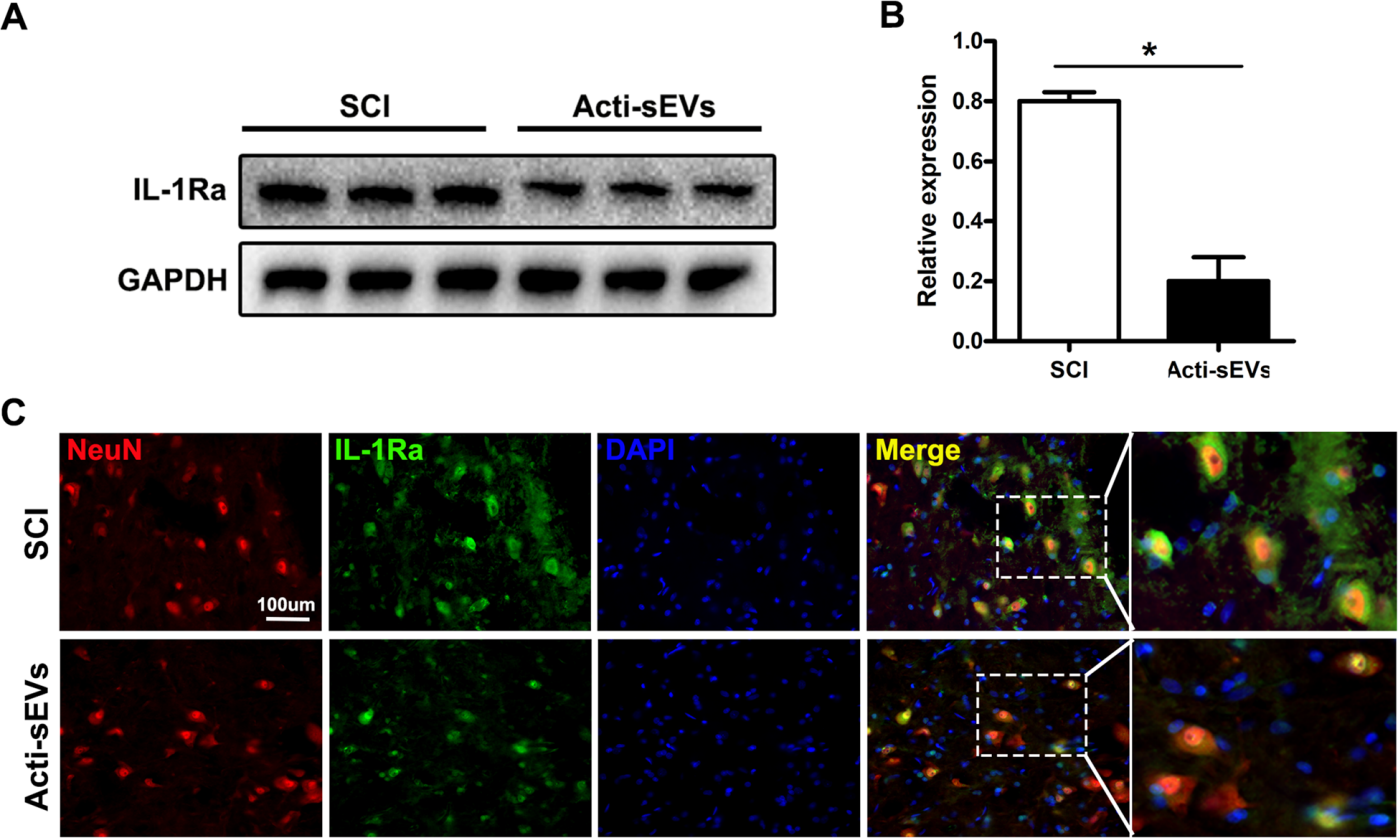
CCL2-induced microglial activation may aggravate neuronal apoptosis. **a**, **b** Western blotting detection of IL-1Ra protein expression in spinal cord tissue. **c** Immunofluorescence detection of IL-1Ra protein expression in neuronal cells

Studies have shown that the release of IL-1β after the activation of microglia/macrophages after epilepsy increases, and the decrease in IL-1β antagonist IL-1Ra can promote neuronal cell death. After SCI, we found that astrocytes were activated and released CCL2-encapsulating sEVs, thereby promoting the activation of microglia, which in turn released large amounts of IL-1β and other inflammatory factors. We hypothesize that after SCI, IL-1β released by activated microglia may also aggravate neuronal apoptosis. Consequently, we detected the expression of IL-1Ra after SCI by western blotting analysis. The expression of IL-1Ra decreased after SCI, and treatment with Acti-sEVs further decreased the expression of IL-1Ra (Fig. 6a, b). The immunofluorescence results further demonstrated that Acti-sEV treatment reduced the expression of IL-1Ra in neuronal cells (Fig. 6c). Therefore, CCL2-induced microglial activation may aggravate neuronal apoptosis.

To further demonstrate that CCL2-induced microglial activation aggravates neuronal apoptosis, we used western blotting analysis and immunofluorescence in vitro to detect the expression of IL-1Ra after Acti-sEV treatment of neuronal cells. Acti-sEV treatment decreased the expression of IL-1Ra, in agreement with the results in vivo (Supplementary Fig. 6A-C). Then, we cultured neuronal cells with Acti-sEV-treated microglia culture medium (CM). TUNEL staining indicated that Acti-sEV-treated microglia culture aggravated the neuronal apoptosis induced by Acti-sEVs+Glu (Supplementary Fig. 6D, E). Western blotting showed that the expression levels of the pro-apoptotic markers Bax and Cleaved caspase-3 were higher, whereas the expression level of the anti-apoptotic protein Bcl-2 was lower (Supplementary Fig. 6F, G), in the Acti-sEV+Glu+CM group than the Acti-sEV group, thus further confirming the results of TUNEL staining.

In summary, the above results indicated that the activation of microglia induced by sEVs encapsulating CCL2 further aggravates neuronal apoptosis.

### The CCR2 antagonist INCB3344 decreases microglial activation and neuronal apoptosis after SCI

**Fig. 7:**
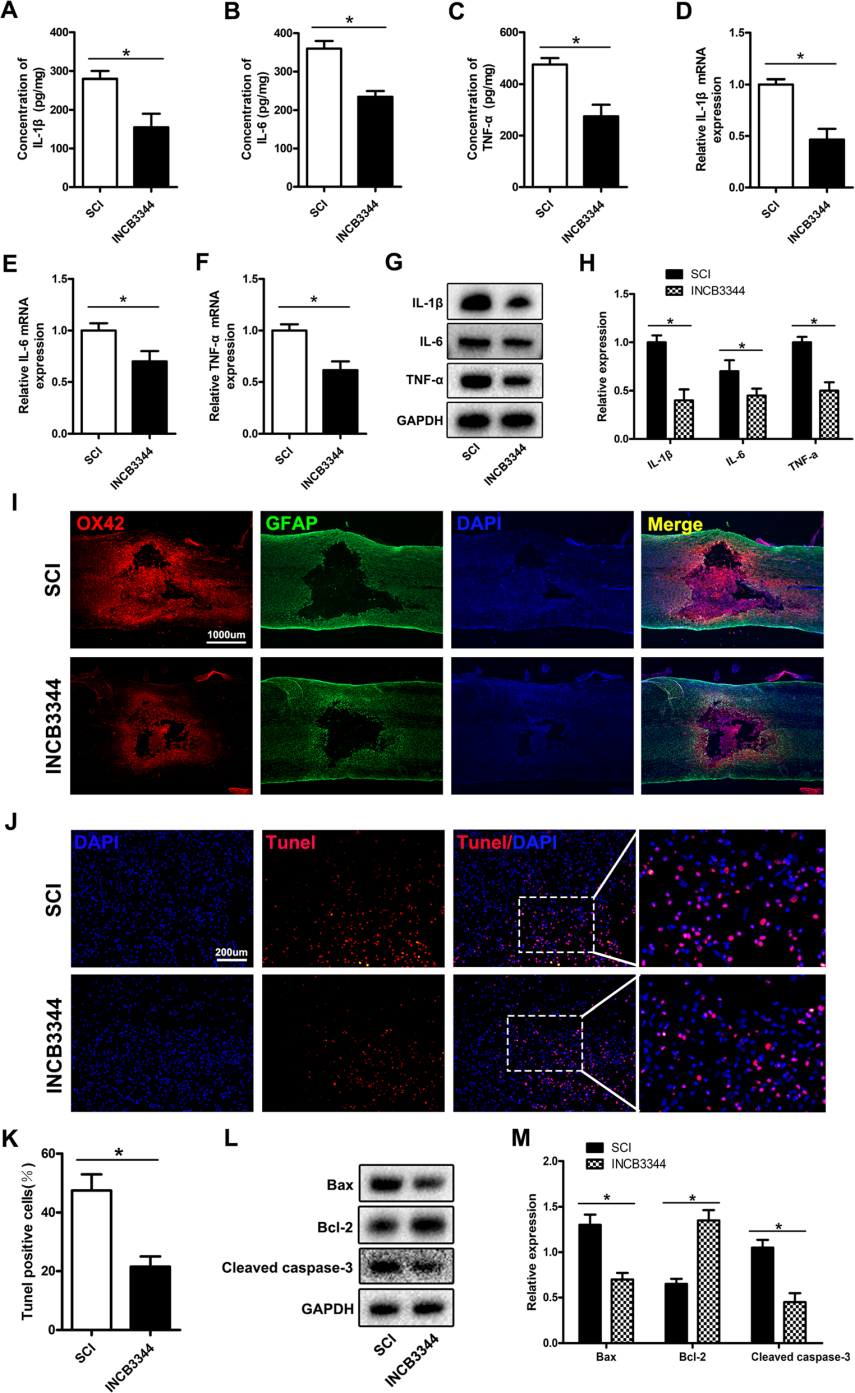
The CCR2 antagonist INCB3344 decreases microglial activation and neuronal apoptosis after SCI. **a–h** ELISA, qRT-PCR, and western blotting evaluation of spinal cord TNF-α, IL-1β, and IL-6 expression. **i** Immunofluorescence staining to detect the expression of the microglial activation marker OX42. **j**, **k** TUNEL staining to detect spinal cord apoptosis. **l**, **m** Western blotting to detect spinal cord apoptosis-related protein expression

We previously demonstrated in vitro that the CCR2 antagonist INCB3344 decreases microglial activation and neuronal apoptosis. To further confirm in vivo that inhibiting the CCL2-CCR2 pathway diminishes the activation of microglia and neuronal apoptosis caused by astrocyte activation, we used INCB3344 (1 mM, 25 μL/rat) to treat SCI rats. ELISA, qRT-PCR, and western blotting were used to detect the influence of INCB3344 on TNF-α, IL-1β, and IL-6 after activation of microglia. The ELISA results showed that, compared with the SCI group, INCB3344 decreased the expression of TNF-α, IL-1β and IL-6 after SCI (Fig. 7a–c), and qRT-PCR and western blotting further confirmed these results (Fig. 7d–h). Immunofluorescence analysis of OX42 showed that INCB3344 inhibited the activation of microglia after SCI, as compared with the SCI group results (Fig. 7i). Then, we used the TUNEL method to evaluate neuronal cell apoptosis in the SCI area and found that INCB3344 inhibited neuronal cell apoptosis, as compared with the SCI group results (Fig. 7j, k). Western blotting showed that, compared with those in the SCI group, the expression levels of the pro-apoptosis-related markers Bax and Cleaved caspase-3 in the INCB3344 group were lower, whereas those of the anti-apoptotic protein Bcl-2 were higher (Fig. 7l, m), thus further confirming the results of TUNEL staining.

In summary, the above results showed that inhibiting the CCL2-CCR2 pathway can block the release of CCL2-encapsulated sEVs after activation of astrocytes, thereby diminishing the effects on microglial activation and neuronal apoptosis.

### INCB3344 promotes the recovery of motor function in SCI rats

**Fig. 8:**
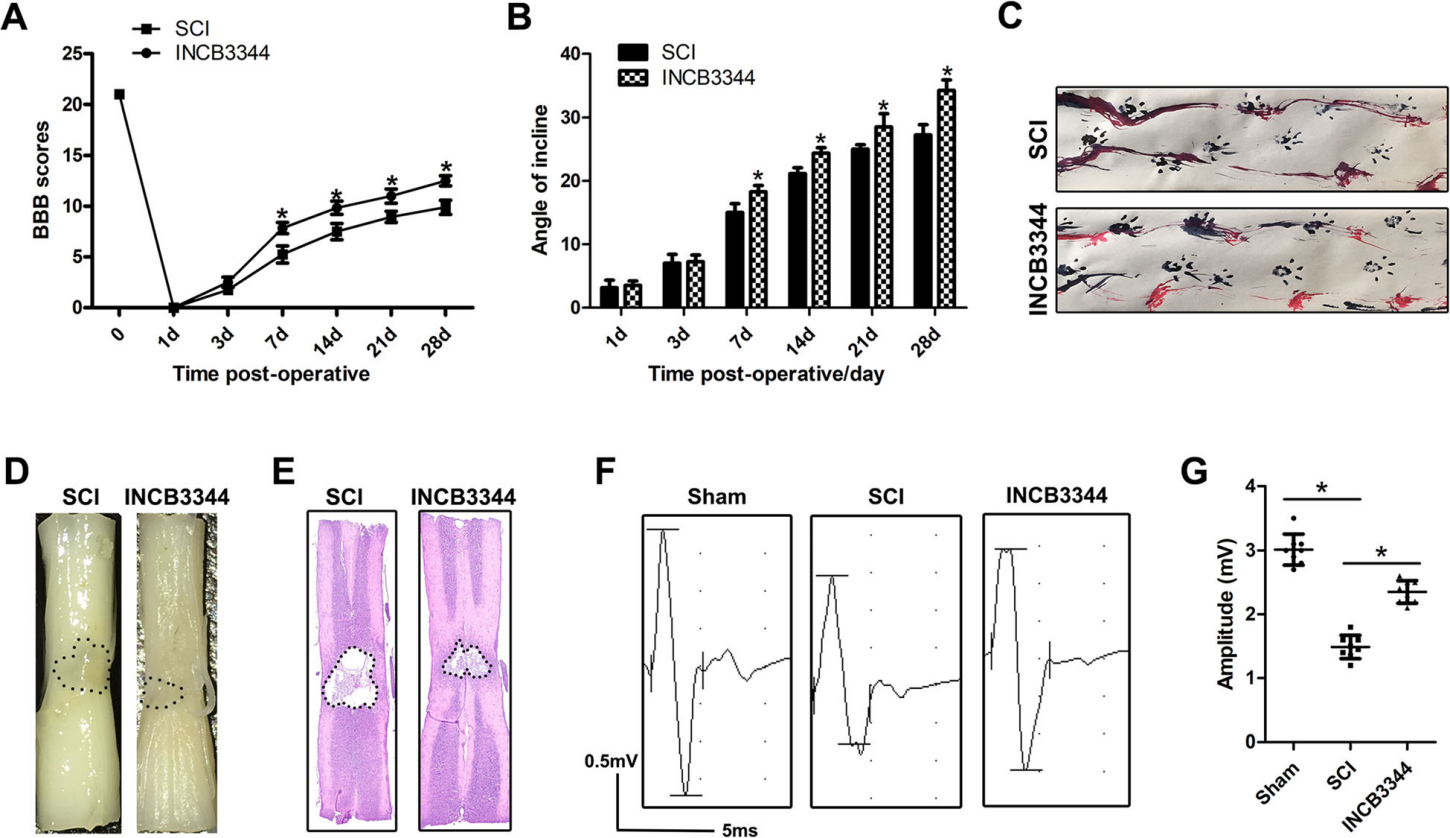
INCB3344 promotes the recovery of motor function in SCI rats. **a**, **b** BBB scores and inclined plane test at different time periods after SCI. **c** Representative footprints of rats walking 28 days after SCI. **d** Gross morphology of the SCI area. **e** Typical HE-stained sagittal section. **f**, **g**. At 28 days after injury, MEP analysis was performed on different groups for electrophysiological evaluation

Next, we further evaluated the effect of the CCL2-CCR2 pathway inhibitor INCB3344 on the recovery of motor function in rats with SCI. The BBB score evaluated the influence of INCB3344 on the recovery of motor function after SCI. At 2–4 weeks after SCI, the BBB score of the INCB3344 group significantly increased (Fig. 8a). The results of the inclined plane test also showed that the maximum inclination angle of the rats in the INCB3344 group was higher than that in the SCI group (Fig. 8b). In addition, we performed a footprint experiment on rats 28 days after SCI. Compared with the SCI group rats, the rats treated with INCB3344 showed a significant improvement in gait recovery and motor coordination (Fig. 8c). As shown in Fig. 8d, the general shape of the injured spinal cord clearly revealed the injured part of the spinal cord. The lesion area after INCB3344 treatment was significantly smaller than that in the SCI group. Next, through HE staining, we found that administration of INCB3344 significantly decreased the area of tissue loss after trauma (Fig. 8e). To further study the recovery of exercise function and behavior, we applied electrophysiological analysis. As shown in Fig. 8f and g, on the 28th day after injury, the MEP amplitude of the INCB3344 group was higher than that of the SCI group, thus indicating that the limbs showed better electrophysiological function recovery after INCB3344 administration.

In summary, these results indicated that inhibiting the CCL2-CCR2 pathway promotes the recovery of functional behavior in rats after SCI.

## Discussion

SCI is a serious disease causing nervous system damage, which can lead to severe motor dysfunction and poor prognosis [30]. Changes in the microenvironment of the injury site lead to a cascade of reactions. Even the neurons that survive the initial traumatic injury may be lost in subsequent pathogenic events such as neuroinflammation and apoptosis. This secondary damage can lead to irreparable damage and loss of function [30, 31]. Changes in the microenvironment after SCI are complex, and studying the interactions among nerve cells is important [32]. In this study, we examined astrocyte activation-mediated microglia and neuronal apoptosis by using an experimental model of acute SCI and performed in vitro cell culture studies. We found that after SCI, activated astrocytes release CCL2, act on microglia and neuronal cells through the sEV pathway, promote neuronal cell apoptosis after binding the CCR2, and simultaneously promote the activation of microglia. Subsequently, the activated microglia release IL-1β, which acts on neuronal cells, thereby further aggravating their apoptosis.

Chemokines and their receptors are widely present in the nervous system and play an important role in regulating cell migration, neurotransmission, and the initiation and maintenance of inflammatory reactions. The chemokine CCL2 is involved in the pathogenesis of many different diseases, including obesity, atherosclerosis, autoimmune diseases, and many neurological diseases [33]. In particular, recent evidence has indicated that CCL2 plays a vital role in the nervous system. Upregulation of CCL2 has been confirmed in animal models of traumatic brain injury, cerebral infarction, and ischemia [34, 35]. In this study, we found that the expression of CCL2 in rats with SCI was higher than that in the normal group. Although most cell types express CCL2, including astrocytes, microglia, neurons, and brain microvascular endothelial cells [36], evidence from recent years indicates that astrocytes are the main source of CCL2 [37, 38]. Our study also showed, through double immunofluorescence staining, that almost all CCL2 expression increased in astrocytes stained with GFAP in the area of SCI. Astrocytes are one of the most abundant cell types in the CNS and are attracting increasing attention as a key regulator of neuroinflammation [2]. Because astrocytes are part of the blood–brain barrier, they first encounter pro-inflammatory signals generated in the peripheral immune compartment even before the blood–brain barrier collapses [39]. Recent studies have shown that sEVs may play important roles in neurodegenerative states or inflammatory processes. Glial molecules may be delivered to microglia or neurons through sEVs and subsequently mediate neuroinflammation and nerve cell damage [40, 41].

sEVs are small vesicles released by cells, and their surface antigens are characterized according to cellular origin [42]. They are released from different types of cells under normal or pathological conditions, and they affect the activity of recipient cells by carrying activity signals. sEVs have received widespread attention because of their potential to transmit host cell genetic information (such as proteins, mRNA, and microRNAs) [43]. Recent studies have shown that cells communicate with neighboring cells or distant cells through sEV-dependent information transmission [44, 45]. Men et al. have identified miR cargos in secreted sEVs and determined their roles in non-cell-autonomous genetic regulation after transfer to astrocytes [46]. Activation of human astrocyte-derived sEVs regulates neuronal uptake, differentiation, and discharge [47]. Our previous studies have demonstrated that sEVs derived from neural stem cells promote autophagy by delivering 14-3-3t protein, thus decreasing neuroinflammation and neuronal apoptosis, and consequently promoting the repair of SCI [16, 48]. Previous studies have shown that the biogenesis and content of sEVs not only depend on their cell source but also are sensitive to cell state and environmental input [49]. sEV release increases during hypoxia, acidic pH, heat shock, and oxidative stress [15, 50, 51]. In this study, we found that in the acute SCI model, the sEVs produced by the injured spinal cord were significantly elevated beyond the levels in normal spinal cord tissue. These data indicate that changes in the microenvironment after white SCI may be a key factor affecting the production of cellular sEVs. sEVs are derived from the endosomal pathway, which involves the formation of multivesicular bodies by the endosomal sorting complex, which is required for transport and release mediated by the Rab27A/B-dependent pathway [26]. However, the mechanism of increased sEV production after SCI requires further study.

The CCL2-CCR2 pathway participates in the pathological process of CNS through the interaction of astrocytes and microglia. Studies have shown that CCL2 derived from stellate cells activates microglia through CCL2-CCR2 signals, thus participating in cognitive dysfunction and neuroinflammation caused by surgery [52]. Results obtained in experimental animal models and human patients have indicated that after status epilepticus, the chemokine CCL2-CCR2 signal induces neuronal cell death through the activation of STAT3 and the production of IL-1β [53]. In fact, Sheehan et al. have revealed that the activation/migration of microglia caused by excitotoxic injury is diminished in CCL2^−/−^ mice [54]. In agreement with the above results, Kim et al. have demonstrated that in experimental autoimmune encephalomyelitis, loss of astrocyte CCL2 leads to a decrease in the activation of microglia [44]. However, these investigations did not study the roles of sEVs in the regulation of microglia by astrocytes through the CCL2-CCR2 pathway. sEVs may play a key role in cell communication through the delivery of RNA, proteins and bioactive lipids [55, 56]. The characteristics of sEVs vary with the function of the host cells and physiological state [43]. Studies have shown that sEVs containing CCL2 in tubular epithelial cells are essential for the inflammation of the tubulointerstitium induced by albumin [57]. In this study, we found that sEVs released from injured spinal cord tissues encapsulated abundant CCL2 released by activated astrocytes. Double immunostaining showed that the main receptor of CCL2, CCR2, was expressed mainly by microglia and neurons at the injury sites, thus indicating that the CCL2-CCR2 pathway is an important astrocyte-microglia-neuron signal after SCI. More importantly, we found that sEVs containing CCL2 are functionally transmitted to microglia and neuronal cells, and after binding CCR2, results in microglial activation and neuronal apoptosis. Therefore, sEVs may be a new carrier through which chemokines coordinate microglia cell activation and neuronal apoptosis. In vitro experiments further demonstrated that astrocytes release large amounts of sEVs encapsulating CCL2 after activation, and these sEVs promote microglial activation and neuronal apoptosis. In vitro inhibition of the expression of astrocytes CCL2 and the release of sEVs alleviated the activation and migration of microglia and the apoptosis of neuronal cells. In vivo and in vitro experiments using CCR2 antagonists also showed a decrease in activation of microglia, as well as apoptosis of neuronal cells, thus promoting the recovery of motor function in rats with SCI.

Neurons are highly specialized cells, which are a basic structural and functional unit of the nervous system, and have roles in sensing stimulation and conducting excitation [58]. Secondary injuries such as neuronal apoptosis after SCI have a severe effect on spinal cord function, thus eventually leading to irreversible damage to the CNS [59]. Many studies have demonstrated that increasing the expression of pro-inflammatory cytokines (IL-1β and TNF-α) in the CNS leads to neuroinflammation and ultimately to neuronal apoptosis [5]. Tian et al. have demonstrated that microglia/macrophage STAT3 is activated after epileptic seizures, thus resulting in production of the inflammatory cytokine IL-1β, thereby leading to neuronal cell death [53]. In addition, after SCI, IL-1β relies on the activity of p38 mitogen-activated protein kinase to induce neuronal apoptosis [60]. In this study, in vivo and in vitro TUNEL staining and western blotting experiments showed that CCL2 released by activated astrocytes promoted the expression of TUNEL-positive cells, and the expression of the pro-apoptotic related markers Bax and Cleaved caspase-3 increased, whereas the expression of the apoptotic protein Bcl-2 decreased. In addition, CCL2 released by activated astrocytes promoted IL-1β release after activation of microglia and further led to neuronal apoptosis. Interestingly, CCR2 antagonists antagonized neuronal cell apoptosis and promoted the recovery of motor function in rats with SCI. Therefore, we hypothesize that CCR2 antagonists directly protect nerve cells on the one hand and may exert their protective effects on neuronal damage, on the other hand, by mediating the pro-inflammatory feedback of microglia.

**Fig. 9:**
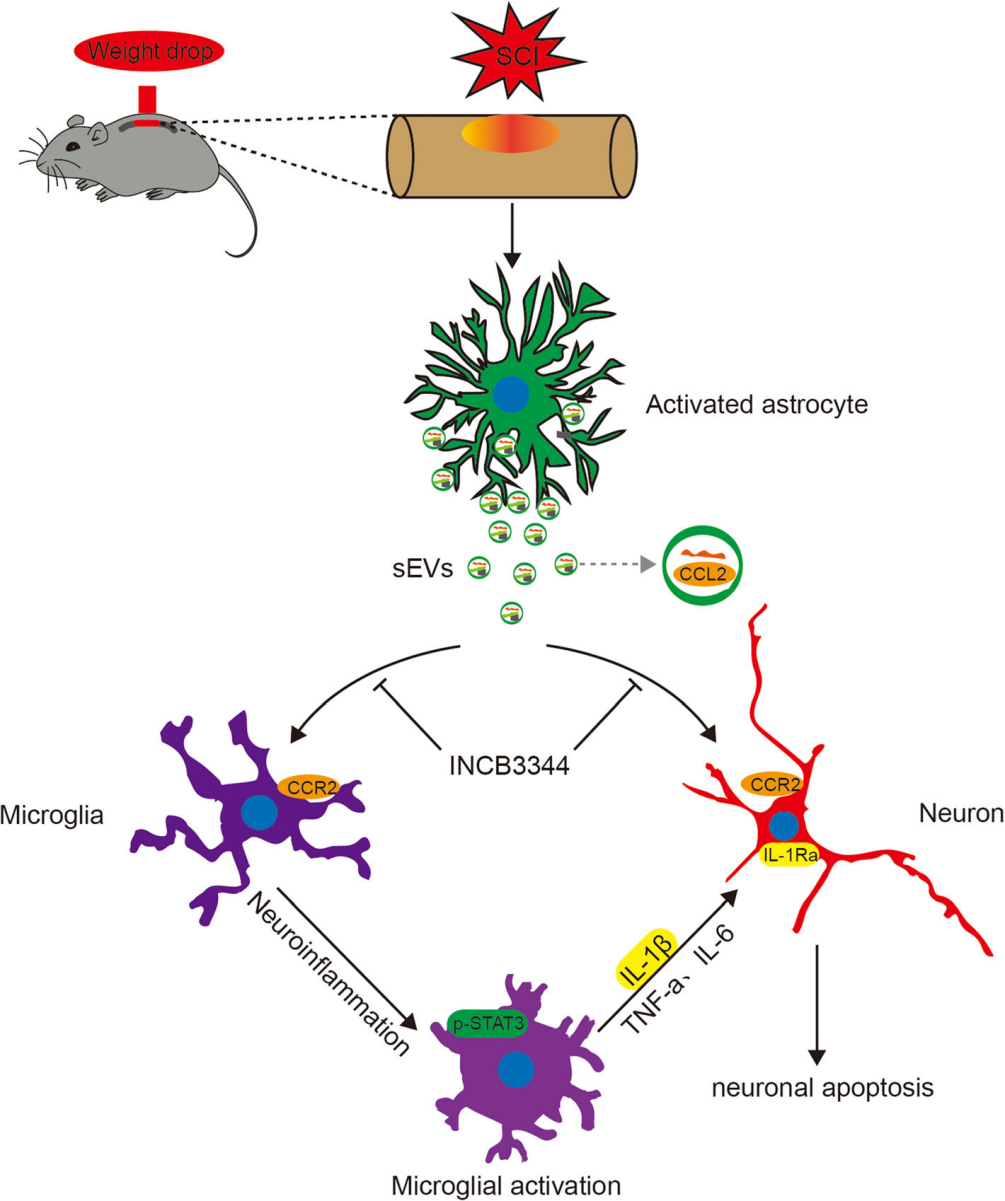
sEVs encapsulating CCL2 from activated astrocytes induce microglial activation and neuronal apoptosis after traumatic spinal cord injury. After SCI, astrocytes activate and release sEVs that encapsulate CCL2, which in turn acts on microglia and neuronal cells through the sEV pathway and binds CCR2, thus promoting neuronal cell apoptosis and the activation of microglia. Subsequently, the activated microglia release IL-1β, which attacks neuronal cells, thereby further aggravating neuronal apoptosis

In summary, we found that, after SCI, astrocytes activate and release large amounts of sEVs that encapsulate CCL2, which in turn acts on microglia and neuronal cells through the sEV pathway and binds CCR2, thus promoting neuronal cell apoptosis and the activation of microglia. Subsequently, the activated microglia release IL-1β, which attacks neuronal cells, thereby further aggravating neuronal apoptosis (Fig. 9).

## Conclusions

In conclusion, the current research aids in the understanding of the dynamic changes in the microenvironment after SCI and clarifies that the interaction of glial cells and neurons is involved in neuroinflammation and spinal cord nerve injury after SCI, thus providing a new theoretical basis of CCL2 as a therapeutic target for SCI.

## Supplementary Information

**Additional file 1: Supplementary Figure 1.** CCL2 expression at different time points after SCI**.** The volcano map of differentially expressed genes in GEO: GSE42828. **Supplementary Figure 2.** Expression and localization of CCR2 after spinal cord injury. **(A, B)** Western blotting detection of CCR2 expression in spinal cord tissue. **(C)** qRT-PCR detection of CCR2 expression in spinal cord tissue. **(D)** FISH detects the location and expression of CCR2 in spinal cord injury tissue. **Supplementary Figure 3.** Identification of primary neurons, microglia and astrocytes. **(A)** NeuN identifies neuron nuclei, and MAP-2 identifies neuron axons. **(B)** CD11b identifies microglia cells. **(C)** GFAP identifies astrocytes. **Supplementary Figure 4.** Inhibiting the release of sEVs from activated astrocytes can reduce microglia activation and neuronal apoptosis. **(A)** Western blotting detection of Rab27a expression in astrocytes. **(B)** Rab27a siRNA can reduce the release of NO from microglia after inhibiting the release of sEVs. **(C, D)** TUNEL staining to detect neuronal apoptosis. **Supplementary Figure 5.** The effects of sEVs containing CCL2 on microglial activation and neuron apoptosis after inhibition of CCR2. **(A-C)** ELISA method to detect TNF-α, IL-1β and IL-6. **(D)** Detection of changes in NO content. **(E-H)** qRT-PCR and western blotting to detect the expression of TNF-α, IL-1β and IL-6. **(I, J)** Transwell assessment of the migration ability of BV2 microglia. **(K-N)** TUNEL staining and western blotting to detect neuronal apoptosis. **Supplementary Figure 6.** Further verification in vitro that CCL2-induced activation of microglia aggravates neuronal apoptosis. **(A, B)** Western blotting detection of IL-1Ra protein expression in neurons. **(C)** Immunofluorescence detection of neurons IL-1Ra protein expression. **(D, E)** TUNEL staining to detect neuronal apoptosis. **(F, G)** Western blotting was used to detect the expression of apoptosis related proteins in neurons.

## Data Availability

Most of the datasets supporting the conclusions of this article are included within this article and the additional files. The datasets used or analyzed during the current study are available on reasonable request.

## Abbreviations

SCI: Spinal cord injury
sEVs: Small extracellular vesicles
BBB: Blood–brain barrier
CNS: Central nervous system
CCL2: C-C Motif Chemokine Ligand 2
CCR2: C-C motif receptor 2
MEP: Motor-evoked potential
Glu: Glutamate
TUNEL: Terminal deoxynucleotidyl transferase dUTP nick end labeling
