# Correction to: Small extracellular vesicles encapsulating CCL2 from activated astrocytes induce microglial activation and neuronal apoptosis after traumatic spinal cord injury

**DOI:** 10.1186/s12974-021-02336-3

**Published:** 2021-12-07

**Authors:** Yuluo Rong, Chengyue Ji, Zhuanghui Wang, Xuhui Ge, Jiaxing Wang, Wu Ye, Pengyu Tang, Dongdong Jiang, Jin Fan, Guoyong Yin, Wei Liu, Weihua Cai

**Affiliations:** Department of Orthopaedics, First Affiliated Hospital of Nanjing Medical University, Nanjing, 210029 Jiangsu China

## Correction to: J Neuroinflamm (2021) 18:196 https://doi.org/10.1186/s12974-021-02268-y

Following publication of the original article [1], the author noticed that Figs. 6 and 7 of the published version of this article were incorrectly uploaded. Presented here are the corrected Figs. 6 and  7.

**Fig. 6:**
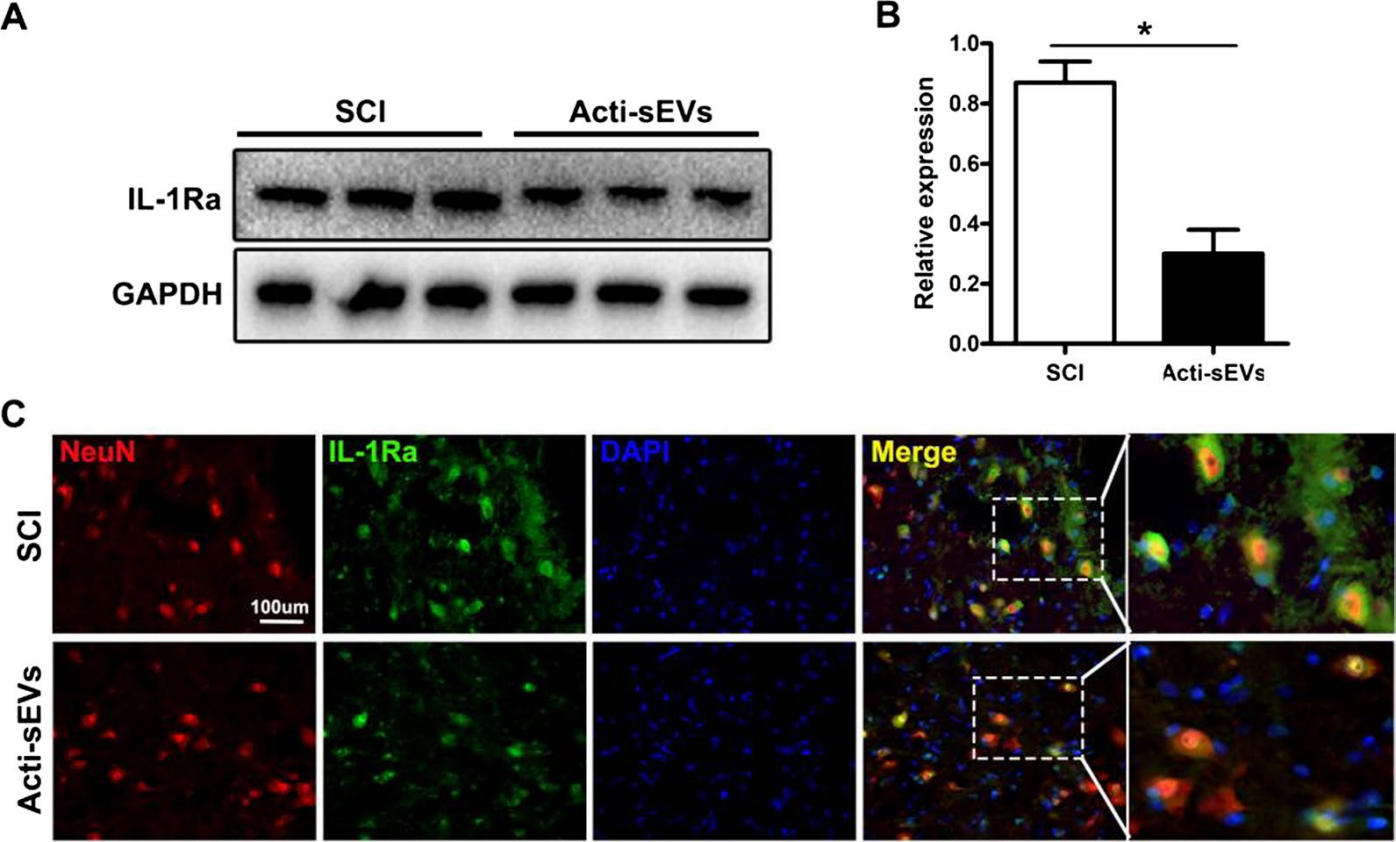
CCL2-induced microglial activation may aggravate neuronal apoptosis. **A**, **B** Western blotting detection of IL-1Ra protein expression in spinal cord tissue. **C** Immunofluorescence detection of IL-1Ra protein expression in neuronal cells

**Fig. 7:**
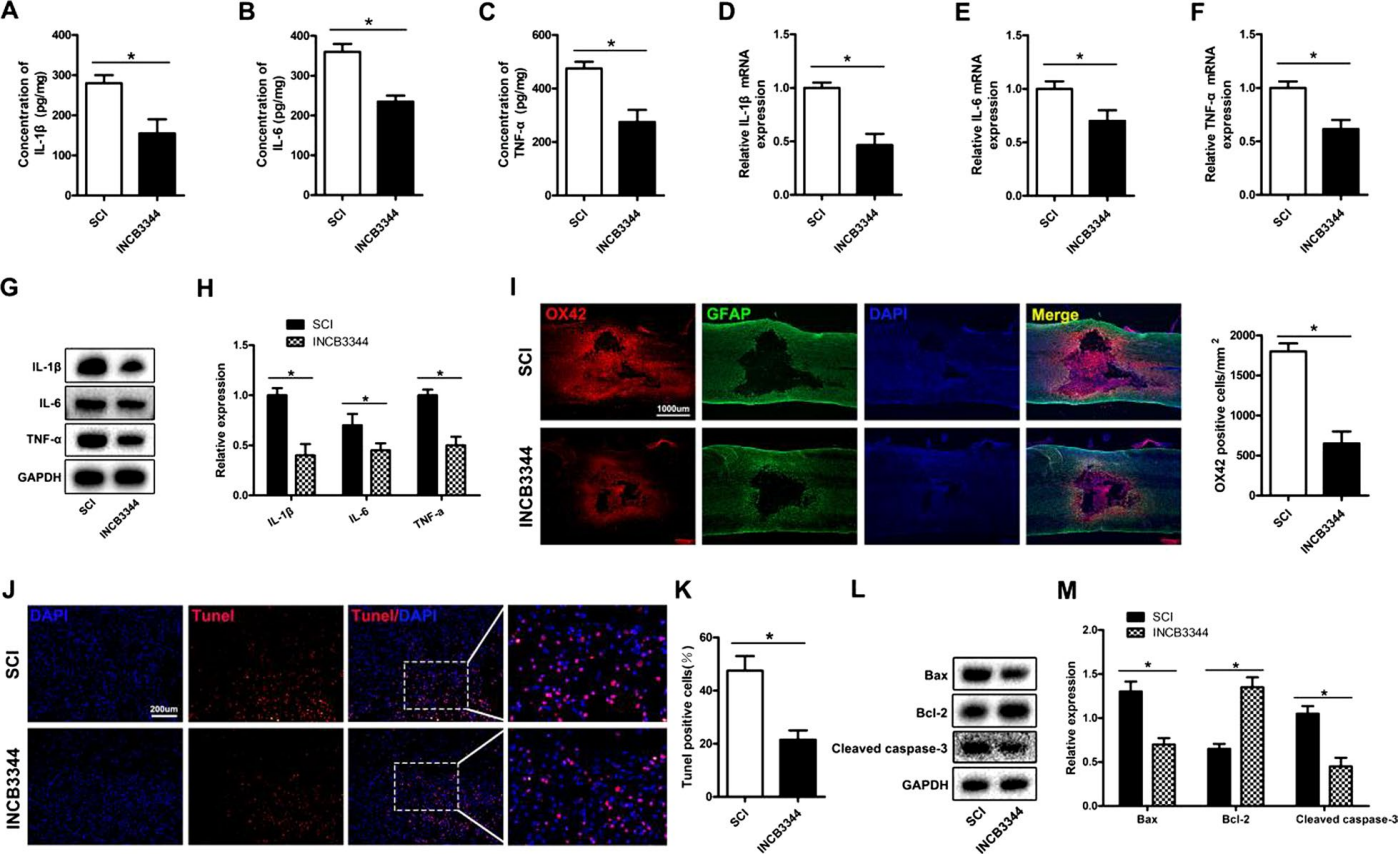
The CCR2 antagonist INCB3344 decreases microglial activation and neuronal apoptosis after SCI. **A–H** ELISA, qRT-PCR and western blotting evaluation of spinal cord TNF-α, IL-1β and IL-6 expression. **I** Immunofluorescence staining to detect the expression of the microglial activation marker OX42. **J**, **K** TUNEL staining to detect spinal cord apoptosis. **L**,** M** Western blotting to detect spinal cord apoptosis related protein expression

## References

[1] Rong Y, Ji C, Wang Z, Ge X, Wang J, Ye W, Tang P, Jiang D, Fan J, Yin G, Liu W, Cai W (2021). Small extracellular vesicles encapsulating CCL2 from activated astrocytes induce microglial activation and neuronal apoptosis after traumatic spinal cord injury. J Neuroinflamm.

