# Supplementary material for: Small extracellular vesicles encapsulating CCL2 from activated astrocytes induce microglial activation and neuronal apoptosis after traumatic spinal cord injury
Source: J Neuroinflammation. 2021 Sep 12;18:196. doi: 10.1186/s12974-021-02268-y (PMC8436564; doi:10.1186/s12974-021-02268-y)
Supplement: Supplementary file 1 — Additional file 1: Supplementary Figure 1. CCL2 expression at different time points after SCI. The volcano map of differentially expressed genes in GEO: GSE42828. Supplementary Figure 2. Expression and localization of CCR2 after spinal cord injury. (A, B) Western blotting detection of CCR2 expression in spinal cord tissue. (C) qRT-PCR detection of CCR2 expression in spinal cord tissue. (D) FISH detects the location and expression of CCR2 in spinal cord injury tissue. Supplementary Figure 3. Identification of primary neurons, microglia and astrocytes. (A) NeuN identifies neuron nuclei, and MAP-2 identifies neuron axons. (B) CD11b identifies microglia cells. (C) GFAP identifies astrocytes. Supplementary Figure 4. Inhibiting the release of sEVs from activated astrocytes can reduce microglia activation and neuronal apoptosis. (A) Western blotting detection of Rab27a expression in astrocytes. (B) Rab27a siRNA can reduce the release of NO from microglia after inhibiting the release of sEVs. (C, D) TUNEL staining to detect neuronal apoptosis. Supplementary Figure 5. The effects of sEVs containing CCL2 on microglial activation and neuron apoptosis after inhibition of CCR2. (A-C) ELISA method to detect TNF-α, IL-1β and IL-6. (D) Detection of changes in NO content. (E-H) qRT-PCR and western blotting to detect the expression of TNF-α, IL-1β and IL-6. (I, J) Transwell assessment of the migration ability of BV2 microglia. (K-N) TUNEL staining and western blotting to detect neuronal apoptosis. Supplementary Figure 6. Further verification in vitro that CCL2-induced activation of microglia aggravates neuronal apoptosis. (A, B) Western blotting detection of IL-1Ra protein expression in neurons. (C) Immunofluorescence detection of neurons IL-1Ra protein expression. (D, E) TUNEL staining to detect neuronal apoptosis. (F, G) Western blotting was used to detect the expression of apoptosis related proteins in neurons. [file 12974_2021_2268_MOESM1_ESM.docx]

**
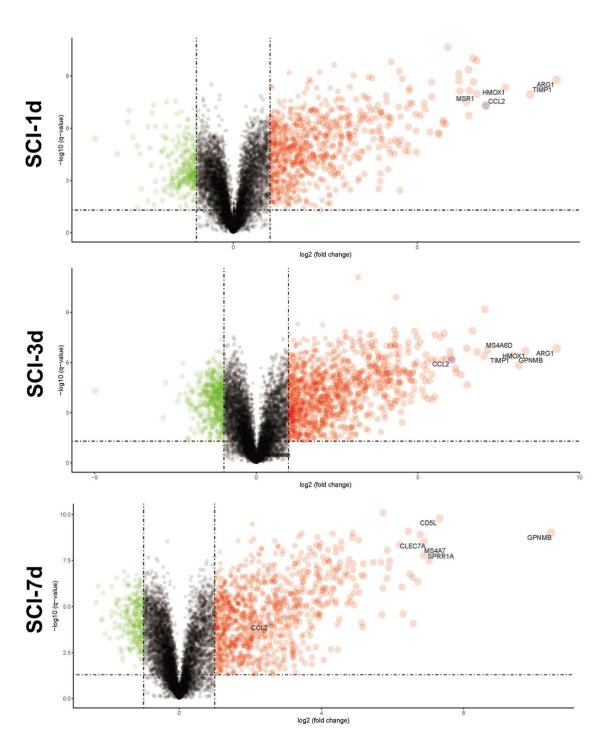
**

**Supplementary Figure 1** **CCL2 expression at different time points after SCI.** The volcano map of differentially expressed genes in GEO: GSE42828.

**
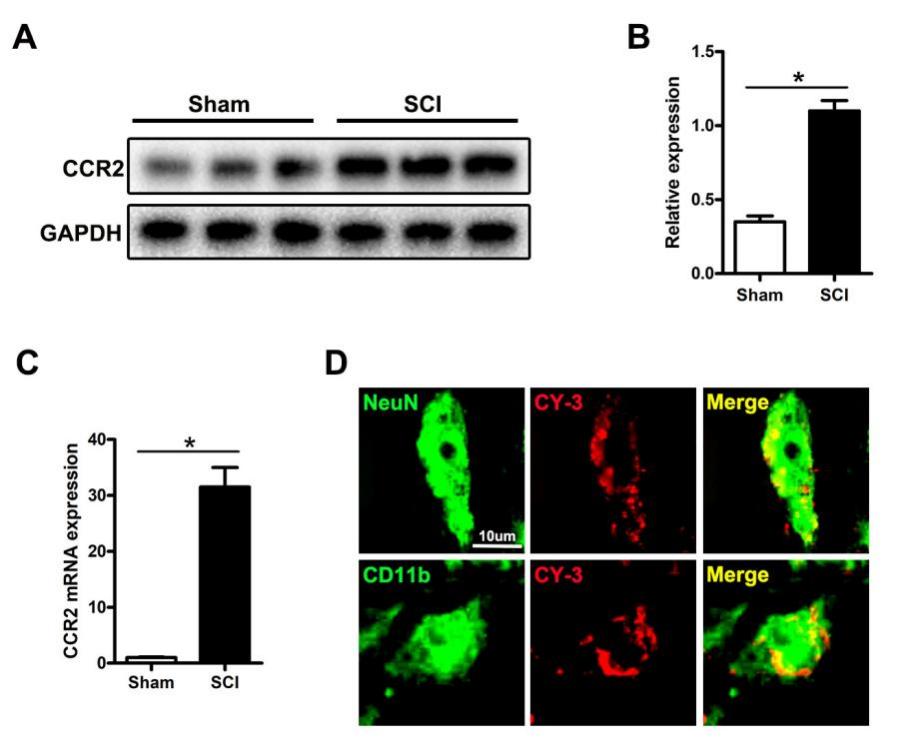
**

**Supplementary Figure 2** **Expression and localization of CCR2 after spinal cord injury. (A, B)** Western blotting detection of CCR2 expression in spinal cord tissue. **(C)** qRT-PCR detection of CCR2 expression in spinal cord tissue. **(D)** FISH detects the location and expression of CCR2 in spinal cord injury tissue.

**
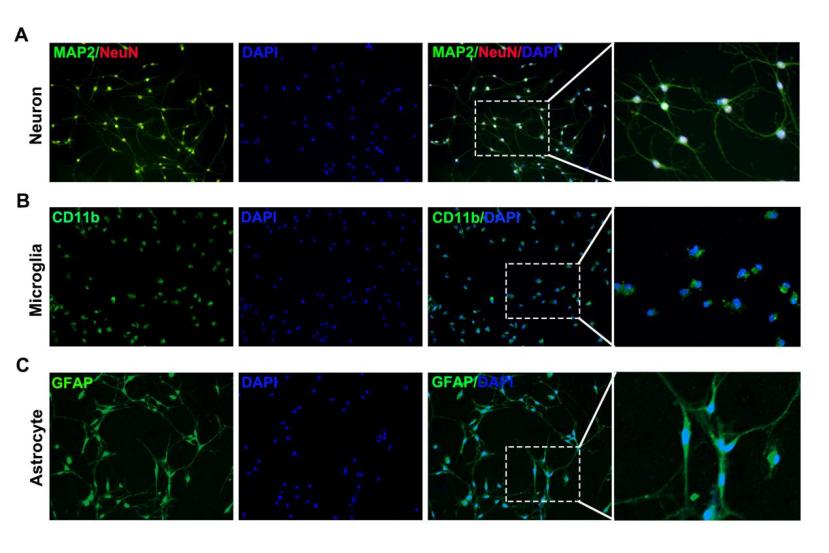
**

**Supplementary Figure 3 Identification of primary neurons, microglia and astrocytes. (A)** NeuN identifies neuron nuclei, and MAP-2 identifies neuron axons. **(B)** CD11b identifies microglia cells. **(C)** GFAP identifies astrocytes.

**
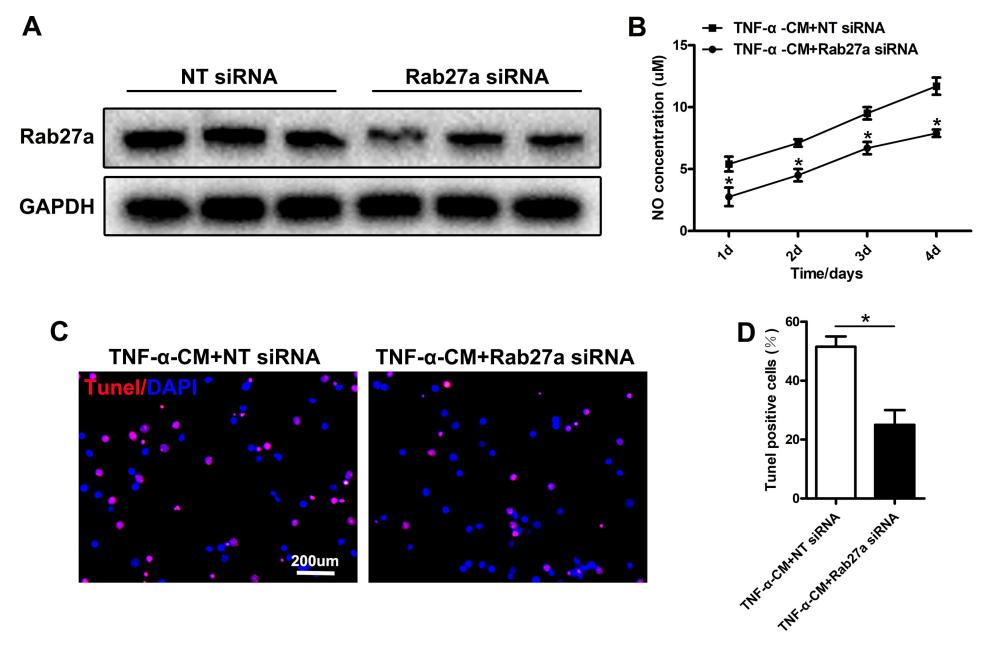
**

**Supplementary Figure 4 Inhibiting the release of sEVs from activated astrocytes can reduce microglia activation and neuronal apoptosis.** **(A)** Western blotting detection of Rab27a expression in astrocytes. **(B)** Rab27a siRNA can reduce the release of NO from microglia after inhibiting the release of sEVs. **(C,D)** TUNEL staining to detect neuronal apoptosis.

**
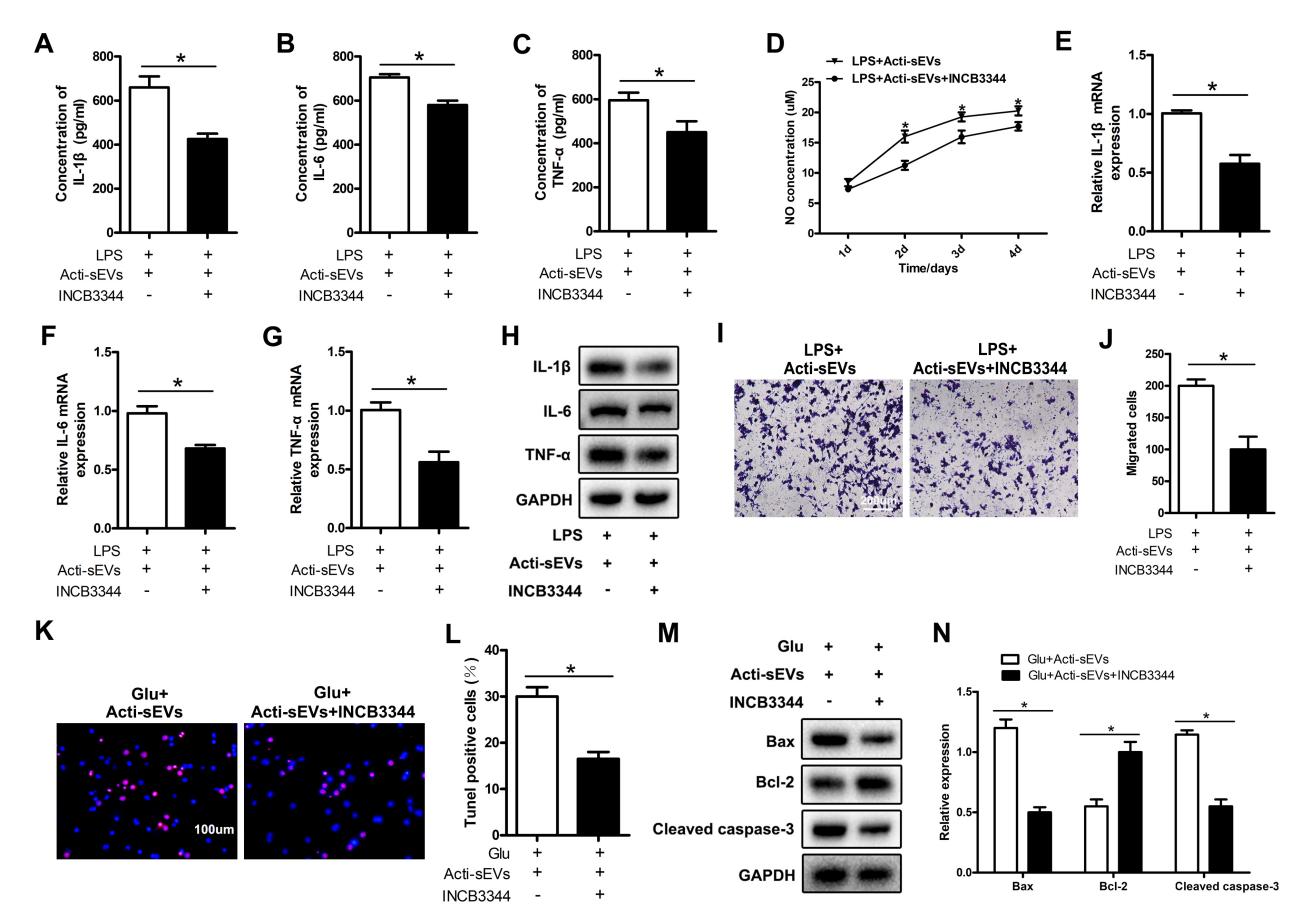
**

**Supplementary Figure 5 The effects of sEVs containing CCL2 on microglial activation and neuron apoptosis after inhibition of CCR2.** **(A-C)** ELISA method to detect TNF-α, IL-1β and IL-6. **(D)** Detection of changes in NO content. **(E-H)** qRT-PCR and western blotting to detect the expression of TNF-α, IL-1β and IL-6. **(I, J)** Transwell assessment of the migration ability of BV2 microglia. **(K-N)** TUNEL staining and western blotting to detect neuronal apoptosis.

**
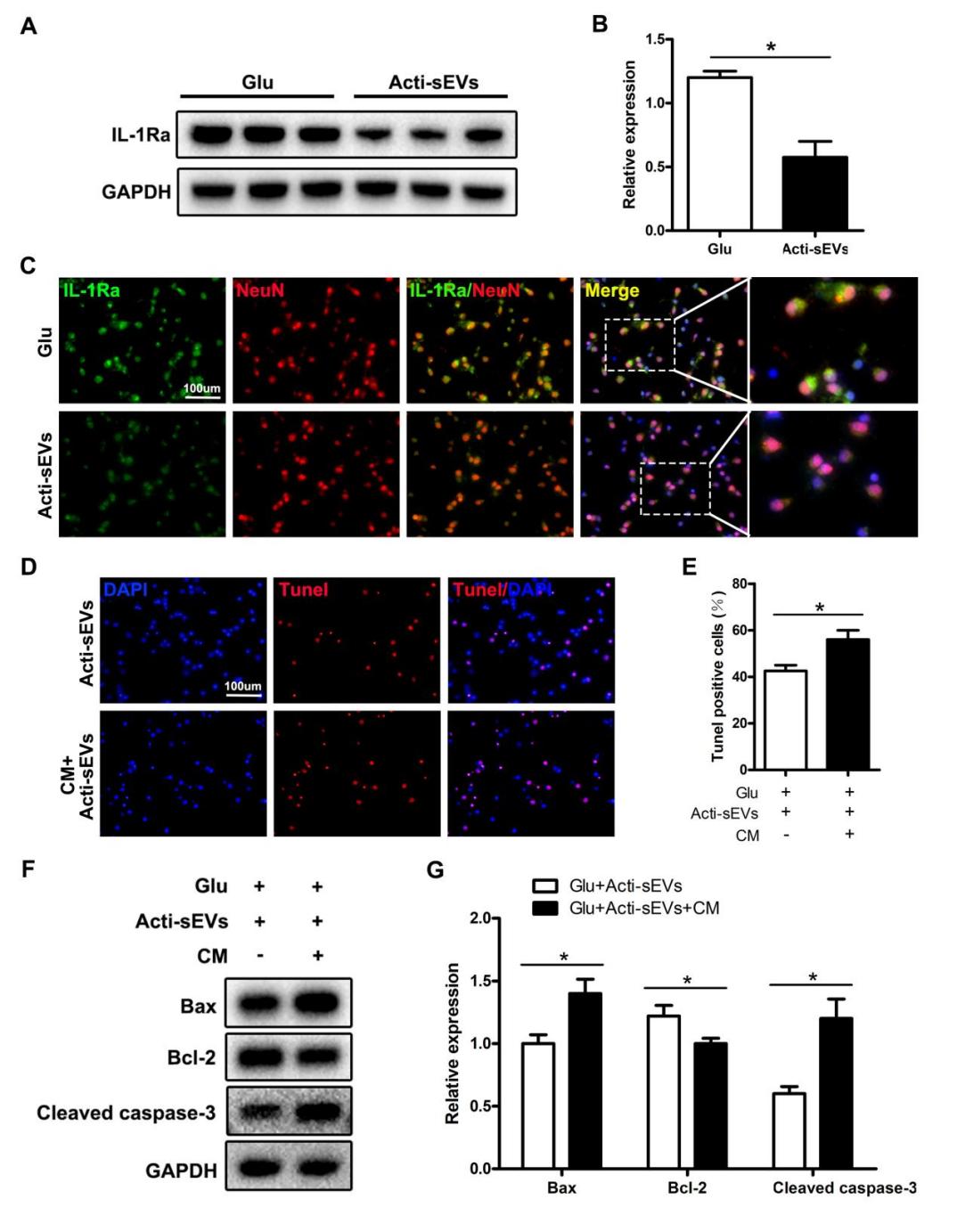
**

**Supplementary Figure 6 Further verification in vitro that CCL2-induced activation of microglia aggravates neuronal apoptosis. (A, B)** Western blotting detection of IL-1Ra protein expression in neurons. **(C)** Immunofluorescence detection of neurons IL-1Ra protein expression. **(D, E)** TUNEL staining to detect neuronal apoptosis. **(F, G)** Western blotting was used to detect the expression of apoptosis related proteins in neurons.
